# The inhibition of lung cancer cell migration by AhR-regulated autophagy

**DOI:** 10.1038/srep41927

**Published:** 2017-02-14

**Authors:** Chi-Hao Tsai, Ching-Hao Li, Yu-Wen Cheng, Chen-Chen Lee, Po-Lin Liao, Cheng-Hui Lin, Shih-Hsuan Huang, Jaw-Jou Kang

**Affiliations:** 1 Institute of Toxicology, College of Medicine, National Taiwan University, Taipei, Taiwan; 2 Department of Physiology, School of Medicine, College of Medicine, Taipei Medical University, Taipei, Taiwan; 3 Graduate Institute of Medical Sciences, College of Medicine, Taipei Medical University, Taipei, Taiwan; 4 School of Pharmacy, College of Pharmacy, Taipei Medical University, Taipei, Taiwan; 5 Department of Microbiology and Immunology, School of Medicine, China Medical University, Taichung, Taiwan

## Abstract

The aryl hydrocarbon receptor (AhR) is a ligand-activated transcription factor that is highly expressed in multiple organs and tissues. Whereas AhR mediates the metabolism of xenobiotic and endogenous compounds, its novel function in cancer epithelial-mesenchymal transition (EMT) remains controversial. Autophagy also participates in tumour progression through its functions in cell homeostasis and facilitates adaptation to EMT progression. In the present study, we found that AhR-regulated autophagy positively modulates EMT in non-small cell lung cancer cells. The motility of A549, H1299, and CL1-5 cells were correlated with different AhR expression levels. Invasive potential and cell morphology also changed when AhR protein expression was altered. Moreover, AhR levels exerted a contrasting effect on autophagy potential. Autophagy was higher in CL1-5 and H1299 cells with lower AhR levels than in A549 cells. Both AhR overexpression and autophagy inhibition decreased CL1-5 metastasis *in vivo*. Furthermore, AhR promoted BNIP3 ubiquitination for proteasomal degradation. AhR silencing in A549 cells also reduced BNIP3 ubiquitination. Taken together, these results provide a novel insight into the cross-linking between AhR and autophagy, we addressed the mechanistic BNIP3 modulation by endogenous AhR, which affect cancer cell EMT progression.

Lung cancer is a widespread cancer with a high mortality rate in both men and women^1^ and contributes to more than one-quarter of the total cancer-related deaths worldwide. Surgical resection can result in a higher cure rate if lung cancer is detected at an early stage. However, metastasis resulting from the invasion of late-stage lung carcinoma significantly lowers the efficacy of therapy^2^. The elucidation of early molecular regulation of lung carcinogenesis may reveal the underlying mechanisms and new markers for early detection or therapeutic targets. The increase in lung cancer deaths reflects the effects of tobacco smoking and air pollution, which are risk factors for cancer development and progression^3^,^4^,^5^. Polycyclic aromatic hydrocarbons (PAHs) are ubiquitous components present in cigarette smoke and air pollution, and are considered to be the most important carcinogens in these complex mixtures^5^. Chemicals such as PAHs exert direct biological effects by binding to AhR, a ligand-activated receptor.

The aryl hydrocarbon receptor (AhR) belongs to a family of basic helix-loop-helix/Per-ARNT-Sim transcription factors, which can form nuclear heterodimers with the AhR nuclear translocator (ARNT) protein. Both phase I and phase II xenobiotic metabolizing enzymes (e.g., cytochrome P450 (CYP1A1, CYP1A2, CYP1B1), and glutathione *S*-transferases) are transactivated when AhR/ARNT is associated with xenobiotic responsive elements^6^,^7^,^8^. An increasing number of studies indicates that AhR plays a novel role in cell migration, proliferation, and chronic inflammation in carcinogenesis^9^,^10^. Although both tumour-suppressor and pro-oncogenic functions have been reported for AhR in different cancers, the relationship between AhR and cancer metastasis remains unclear.

Invasion and migration are common cancer cell characteristics, and epithelial-mesenchymal transition (EMT) is considered to be a crucial step in the cancer metastatic cascade. During EMT, cells lose their cell–cell junctions via changes in cadherins or other EMT markers, such as E-cadherin downregulation and N-cadherin upregulation^11^,^12^,^13^. These changes in cell morphology and function are accompanied by vimentin and snail protein expression^14^,^15^. Although aberrant EMT is known to contribute to cell motility during carcinoma progression, regulation of the molecular mechanisms involved has not been thoroughly investigated.

Autophagy dysregulation likely differs in cancer pathogenesis, which also has both protective and potentially deleterious processes during different stages^16^. Emerging evidence suggests that autophagy has complex functions in tumour progression and the promotion of cancer cell death^17^. Furthermore, EMT has been observed to be involved in the activation of several important pathways during autophagy^18^. However, the relationship between autophagy and EMT contributing to cancer cell migration remains largely unknown. Although the AhR has been reported to modulate EMT, the results of previous studies are controversial. In this study, we found that AhR protein expression altered EMT via cell autophagy in different lung cancer cells and that high endogenous AhR expression contributed to downregulation of cell autophagy and EMT inhibition. We sought to elucidate the exact signalling pathway involved in AhR-regulated autophagy and EMT, particularly the novel crosstalk to determine how AhR levels affect lung cancer metastasis.

## Results

### Low expression of AhR protein contributes to higher cell migration in non-small cell lung cancer (NSCLC)

**Figure 1:**
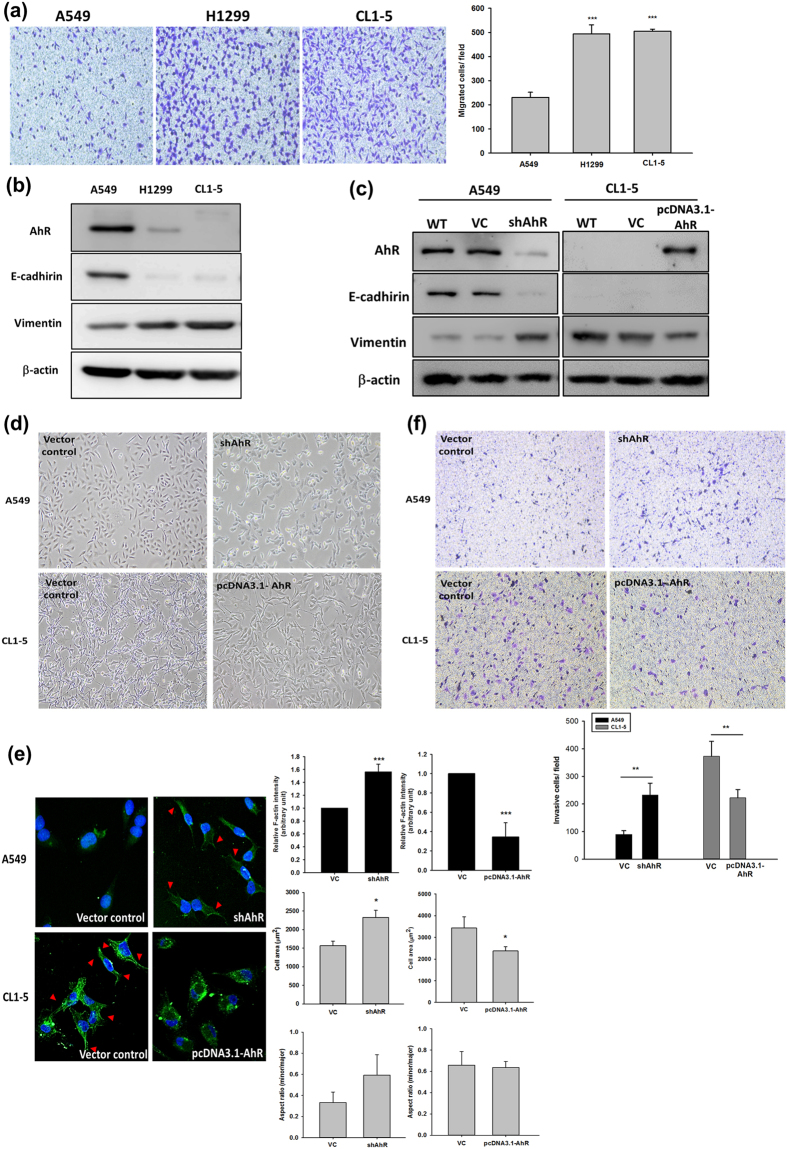
Aryl hydrocarbon receptor (AhR) expression in different cells was correlated with E-cadherin and vimentin expression and cell motility. Protein expression was evaluated by western blotting. Cells (10^4^ cells/Transwell) were seeded on matrigel-coated Transwell inserts and incubated for 16 h. The migrated/invasive cells were stained with crystal violet and counted using the Image-Pro plus software. Images were acquired at 40× magnification. (**a**) Cell migration potential between A549, H1299, and CL1-5 cells. (**b**) Expression of AhR, E-cadherin, and vimentin in A549, H1299, and CL1-5 cells. Full-length blots are presented in Supplementary Fig. S11. (**c**) AhR silencing and overexpression in A549 and CL1-5 cells, respectively, and vimentin expression but not E-cadherin was also affected. Full-length blots are presented in Supplementary Fig. S12. (**d**,**e**) Cell morphology was altered in A549 and CL1-5 cells. Immunostaining of F-actin (green fluoresce) was observed by confocal microscope. The fluorescence intensity (a.u.), cell area and minor/major axis ratios were also measured by Image Pro^TM^ and data analysed as indicated. Arrowheads indicate cells expressing the actin stress fibers. (**f**) And the cell invasive potential were altered in A549 and CL1-5 cells. The quantified data were analysed as cell number per field and expressed as the mean ± SD from three independent experiments. **P < 0.01; ***P < 0.001 compared to the control group.

AhR expression is an important parameter in carcinogenesis and tumour metastasis under physiological conditions^10^. The limited number of studies of AhR protein expression in cancer patients revealed different AhR expression levels in various tumours^19^,^20^. In this study, we observed varying cell motility among human non-small cell lung cancer cell lines. Interestingly, AhR protein expression was correlated with cell migration ability. We also confirmed the protein expression of the EMT markers vimentin and E-cadherin. The results showed that A549 cells, which express high levels of AhR, exhibited lower cell migration ability along with high E-cadherin and low vimentin expression. In contrast, low AhR protein expression in H1299 and CL1-5 cells was associated with higher cell migration ability with increasing vimentin but not E-cadherin protein expression (Fig. 1a and b). We also confirmed the differences in cell invasive potential between NSCLC cells; after comparing the protein expression levels of AhR, A549 and CL1-5 cells were selected for subsequent experiments (Fig. S1). To determine whether the level of AhR is a key factor affecting EMT, AhR was silenced and overexpressed in A549 and CL1-5 cells, respectively, using lentivirus infection systems to observe the changes in EMT protein marker expression and cell motility. AhR knockdown significantly downregulated E-cadherin and increased vimentin protein expression in A549 cells; in contrast, AhR-overexpression decreased vimentin, but did not reverse E-cadherin protein expression in CL1-5 cells (Fig. 1c). Cellular morphology was changed in both AhR-silenced A549 cells and AhR-overexpressing CL1-5 cells. The cell shapes were affected by changing AhR protein level in these two lung cancer cells. High AhR protein level contribute to cells which similar to cobblestone shape; conversely, cells were undergoing mesenchymal-like and elongated spindle shape with low AhR level (Fig. 1d). F-actin cytoskeleton is essential for regulation of cell shape change and motility^21^, which also involve in cytoskeletal reorganisation during EMT^22^. To analyse the cytoskeleton in different AhR expression level cells, we next stained A549 and CL1-5 cells with antibodies to F-actin in the presence or absence of AhR expression, respectively. F-Actin fluorescence intensity was observed by immunostaining. In contrast to high-expressed AhR cells, the stress fiber present in low-expressed AhR cells was observed. Similarly, the trend of cell spreading area have also been revealed. As for corroborating the enlarged cell area in low-expressed AhR, the minor/major axis ratio was quantified (Fig. 1e). Moreover, cell invasion ability was enhanced in AhR-silenced A549 cells and decreased in AhR-overexpressing CL1-5 cells (Fig. 1f). These results suggest that the protein expression level of AhR regulates cellular EMT and that AhR regulates tumour metastasis in lung cancer cells.

### Endogenous AhR protein level affects autophagy-related protein expression associated with cell motility in NSCLC

**Figure 2:**
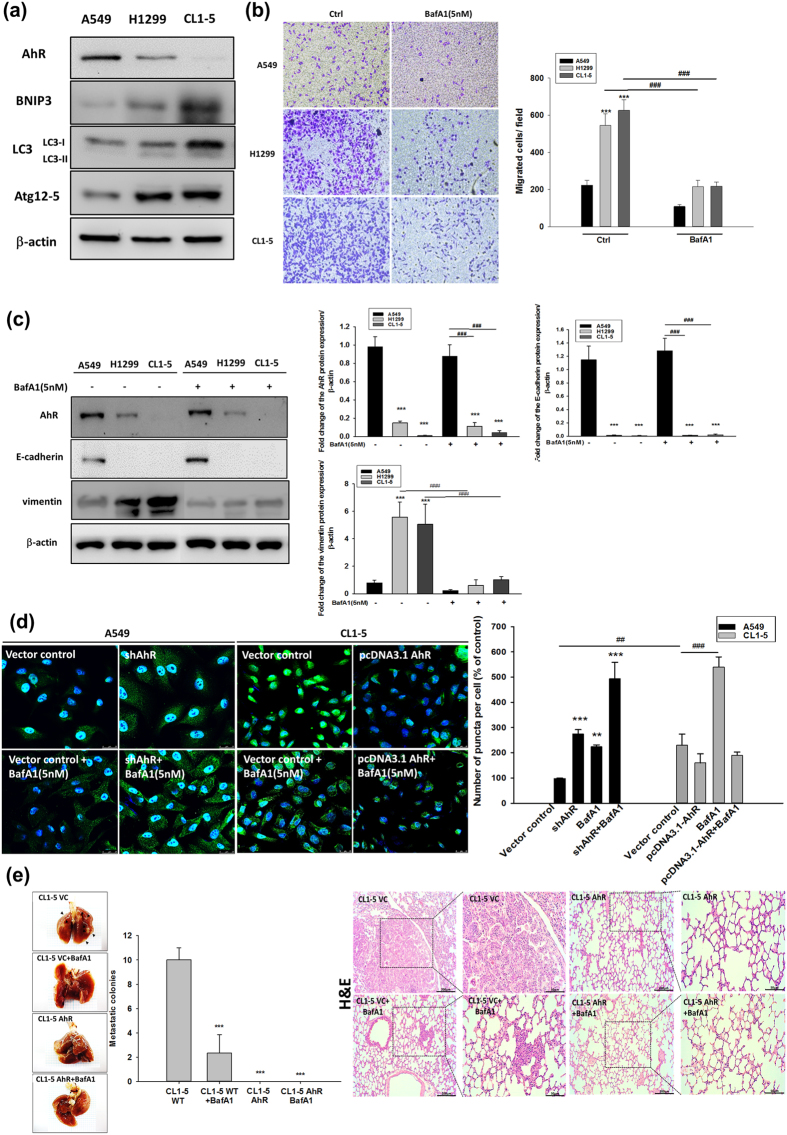
Level of autophagy-related protein expression in different cells was correlated with their cell motility. Protein expression was evaluated by western blotting. Cells (10^4^ cells/Transwell) were seeded on matrigel-coated Transwell inserts, and incubated for 16 h. The migrated cells were stained with crystal violet and images were acquired at 40× magnification. (**a**) Protein expression of AhR, BNIP3, LC3, and ATG12-5 in A549, H1299, and CL1-5 cells. Full-length blots are presented in Supplementary Fig. S13. (**b**) Cell migration potential analysed by BafA1 treatment (5 nM) compared to the control among A549, H1299, and CL1-5 cells. (**c**) Protein expression of AhR, E-cadherin, and vimentin by BafA1 (5 nM) treatment of A549, H1299, and CL1-5 cells. Full-length blots are presented in Supplementary Fig. S14. (**d**) Immunofluorescence microscopy of LC3B (green) in A549 and CL1-5 cells following transfection with shAhR and pcDNA3.1-AhR vector. Nuclei were visualized by staining with Hoechst 33258 (blue). The number of puncta were quantified by Image Pro^TM^, data were presented as percentages normalized to the control (100%). (**e**) Multifocal, well-demarcated, metastatic foci (arrow) were detected in the lung, there were no tumour detected in AhR and AhR + BafA1 groups. The metastatic tumour cells grew in a nest or sheet pattern and showed areas of glandular differentiation and papillary architecture (HE staining). The quantified data were analysed and expressed as the mean ± SD from three independent experiments. **P < 0.01; ***P < 0.001 compared to the control group.

Increasing autophagy in tumour cells is a physiological survival mechanism against antitumor therapy^23^. A previous study reported that autophagy activation may be accompanied by cell EMT progression^24^, but the biphasic mechanism between these processes remains unclear. To determine whether AhR regulates EMT progression via cell autophagy, we found that the autophagy-related markers LC3, ATG12-5, and BNIP3 were highly expressed in cells with low AhR protein expression, particularly in CL1-5 cells (Fig. 2a). Based on AhR protein expression for different migration potentials, we found that the treatment with bafilomycin A1 (BafA1), an inhibitor of late-phase autophagy (5 nM), inhibited A549, H1299, and CL1-5 cell migration (Fig. 2b). Additionally, CL1-5 cells were treated BafA1 in dose-dependent manner (1 nM and 5 nM), which significantly decreased cell migration in high dose group but not low dose group. In contrast to BafA1 treated group, treatment with ATG12 siRNA and BNIP3 knockdown in cells also impaired CL1-5 migration potential (Fig. S2). BafA1 also decreased vimentin expression but did not increase E-cadherin expression in CL1-5 cells (Fig. 2c). Immunofluorescence staining showed that LC3 puncta were increased by BafA1 treatment in AhR-silenced A549 cells compared to wild-type A549 cells; in contrast, AhR-overexpressing CL1-5 cells showed reduced LC3 puncta compared to wild-type CL1-5 cells (Fig. 2d). The transmission electron microscopy also demonstrated reduction of autophagosome in AhR-overexpressing CL1-5 cells where compared to wild-type CL1-5 cells (Fig. S3b). Moreover, *in vivo* tumour metastasis was assessed using wt-A549, and shAhR-A549, wt-CL1-5, and AhR-overexpressing CL1-5 cells by intravenous tail vein injection into mice. wt-CL1-5 with low AhR protein expression showed highly metastatic spread to the lungs, which could be decreased by BafA1 treatment. In contrast, no tumour formation was observed from cells overexpressing AhR alone and combined with BafA1 treatment. As observed on haematoxylin and eosin (HE)-stained sections, metastatic tumour cells grew in a nest or sheet pattern and showed areas of glandular differentiation and papillary architecture (wt-CL1-5). Immunohistochemistry analysis revealed higher BNIP3 expression in the wt-CL1-5 tumours than in BafA1-treated-CL1-5 tumours or normal mouse lung (Fig. S6), confirming that cell lines with low AhR continue to exhibit high expression of autophagy-related proteins *in vivo*. AhR knockdown was also detected in the small metastatic foci of A549 cells in the lung, either (Fig. S5). Taken together, these findings suggest that low levels of AhR not only promote autophagy and migration *in vitro*, but also metastatic tumour formation *in vivo*.

**Figure 3:**
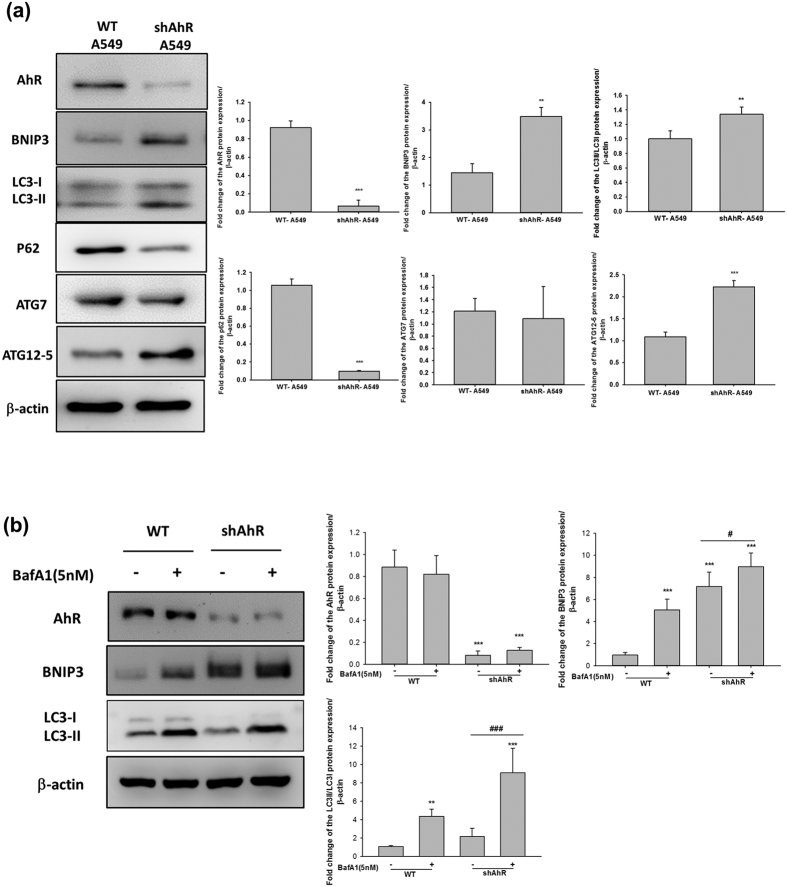
AhR-knockdown increased autophagy in A549 cells. A549 cells were transfected with AhR shRNA (provided by RNAicore) using lentivirus infection for 72 h. Cellular proteins were then isolated for western blottin and autophagy-related proteins were analysed. (**a**) Western blots showed that AhR knockdown increased the protein expression of BNIP3, ATG12-5, and LC3II/LC3I ratio but decreased p62 expression. (**b**) BafA1 (5 nM) treatment of wild-type and AhR knockdown cells for comparison of autophagy flux. Quantification of western blot data showing the fold-change in BNIP3, p62, ATG12-5, LC3, and ATG7 expression. Both BNIP3 and p62 were decreased, whereas ATG12-5 and LC3 were enhanced by AhR knockdown in A549 cells. Full-length blots are presented in Supplementary Fig. S15. The quantified data were analysed and expressed as the mean ± SD from three independent experiments. **P < 0.01; ***P < 0.001 compared to the control group. ^###^P < 0.001 indicate a statistical difference between shAhR groups.

To investigate whether autophagy flux is under AhR-dependent regulation, we measured the levels of related autophagy markers. In accordance with our above results, AhR knockdown increased the levels of BNIP3, LC3II/I, and ATG12-5 proteins. p62 protein expression was dramatically decreased in AhR knockdown cells, indicating the potent induction of autophagy^25^. Interestingly, ATG7 protein expression remained unchanged in AhR-silenced A549 cells (Fig. 3a). BafA1 treatment was used to confirm autophagy flux in AhR knockdown A549 cells compared to in wt-A549 cells. BafA1 treatment resulted in large accumulation of BNIP3 and LC3II/I proteins, even in AhR-silenced A549 cells (Fig. 3b). In addition, the observation of autophagy related marker, LC3 and BNIP3 mRNA expression level were not changed both in A549 and CL1-5 cells (Fig. S7). Additionally, we also investigated the other EMT marker if affected by AhR expression. The results include β-catenin and snail protein/mRNA expression in AhR overexpressed CL1-5 cells. None of detected gene were changed in mRNA patterns, with an exception of AhR, which was overexpressed by overexpressing vector. These result suggest that AhR expression may modify autophagy flux and EMT protein levels rather than genomic regulation.

**Figure 4:**
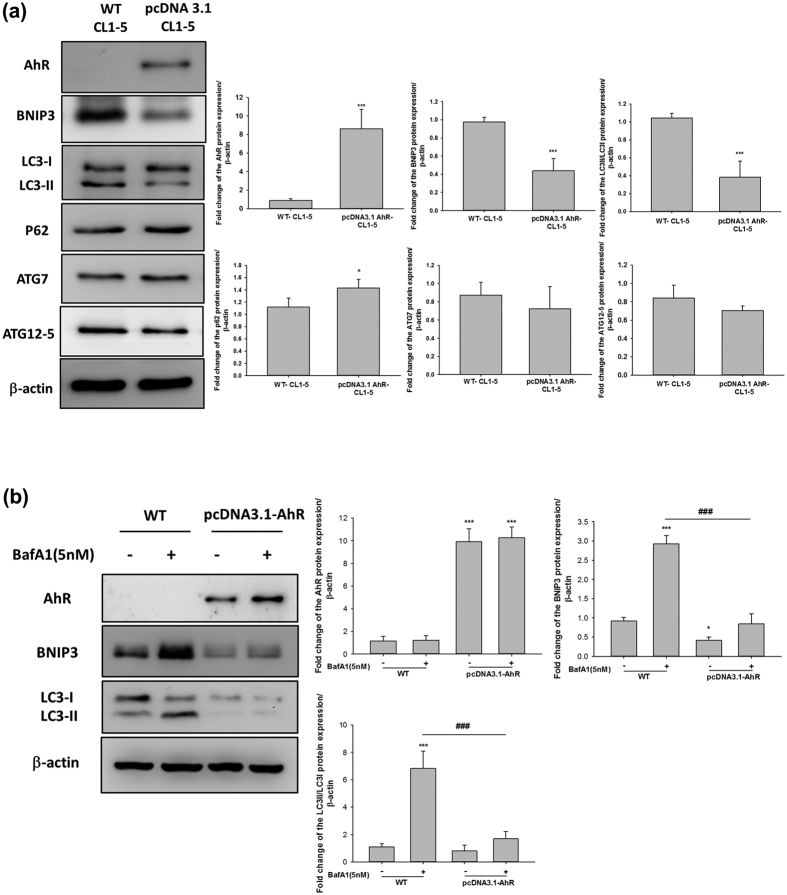
AhR-overexpression decreased autophagy in CL1-5 cells. CL1-5 cells were transfected with pcDNA3.1-AhR for 24 h and autophagy-related proteins were then analysed by western blotting. (**a**) Western blotting showed that AhR overexpression decreased the levels of BNIP3 and LC3II/LC3I ratio. (**b**) BafA1 (5 nM) treatment of wild-type and AhR-overexpressing cells to compare autophagy flux. Quantification of the western blot data showing the fold-changes in BNIP3, p62, ATG12-5, LC3, and ATG7 expression. Full-length blots are presented in Supplementary Fig. S16. The data were analysed and expressed as the mean ± SD from three independent experiments. *P < 0.05; **P < 0.01; ***P < 0.001 compared with the control group. ^###^P < 0.001 indicate a statistical difference between wild-type and AhR overexpression cells in BafA1 treatment groups.

As described above, we also investigated the effect of AhR overexpression on the activation of autophagy. CL1-5 cells overexpressing AhR showed reduced protein expression levels of BNIP3 and ratio of LC3II/I; p62, ATG7, and ATG12-5 were unaffected in these cells, suggesting that autophagy flux is impaired by AhR overexpression (Fig. 4a). BafA1 treatment resulted in the greater accumulation of BNIP3 and LC3II/I proteins in wt-CL1-5 rather in AhR-overexpressing CL1-5 cells (Fig. 4b). We also investigated whether cellular autophagy in AhR-overexpressing H1299 cells was consistent with that in AhR-overexpressing CL1-5 cells. AhR-overexpressing H1299 cells showed changes in BNIP3 and LC3II/I protein expression, which was decreased in AhR-overexpressing cells (Fig. S8). These data indicated that AhR protein levels affect cellular autophagy and support the results of the autophagy flux experiment using BafA1 treatment. Taken together, these data suggest that the differences in cell motility based on AhR levels is mediated by autophagy, which involves crosstalk between AhR and EMT in lung cancer cells.

### BNIP3 is a critical mediator of AhR-regulated autophagy

**Figure 5:**
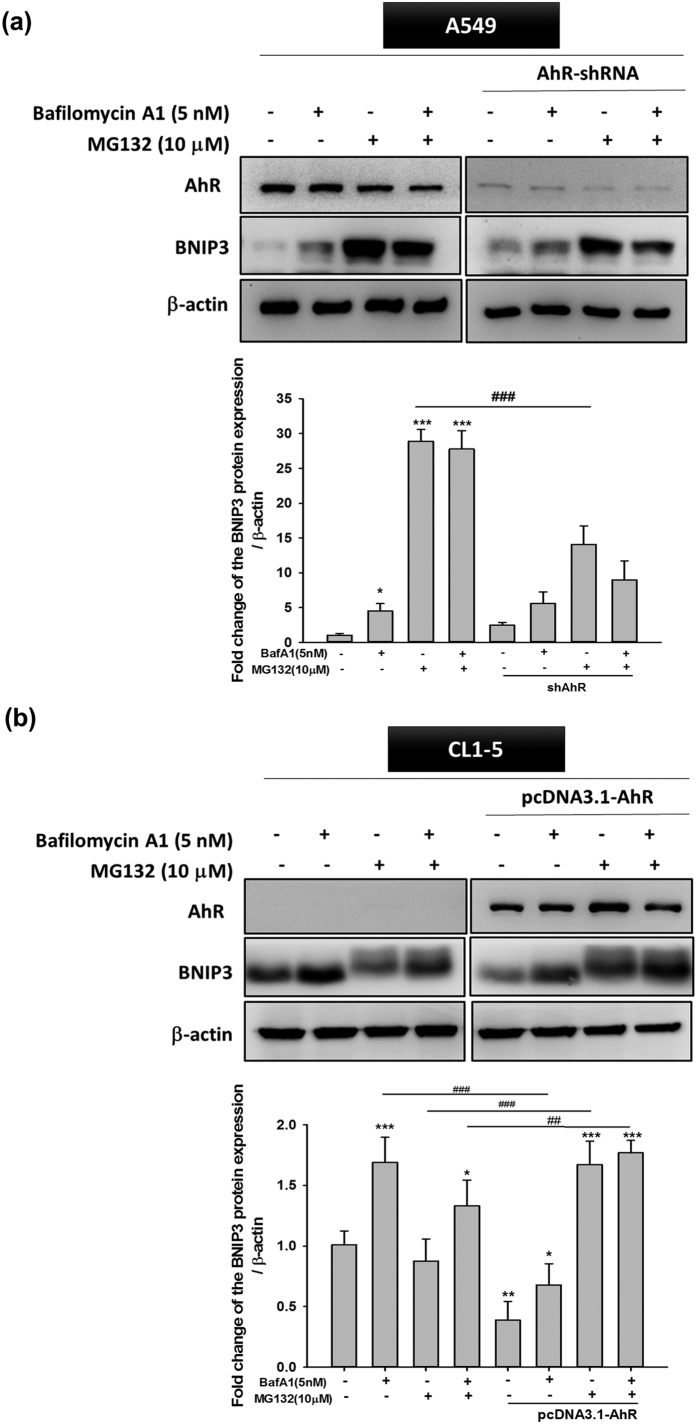
BafA1 and MG132 treatment reversed BNIP3 degradation modulated by AhR in A549 and CL1-5 cells. (**a**) A549 cells were transfected with shAhR and (**b**) CL1-5 were transfected with pcDNA3.1- AhR, respectively for 24 and 72 h, followed by treatment with BafA1 (5 nM) and MG132 (10 μM) for 6 h. Western blotting showed that BafA1 (5 nM) and MG132 (10 μM) inhibited BNIP3 degradation both in AhR-silenced and overexpressing cells. Quantification of the western blot data showed the fold-change in BNIP3. Full-length blots are presented in Supplementary Fig. S17. Quantification was performed by densitometry analysis and expressed as the mean ± SD from three independent experiments. *P < 0.05; **P < 0.01; ***P < 0.001 compared to the control group. ^##^P < 0.01; ^###^P < 0.001 indicate a statistical difference between comparative groups.

Several studies have indicated that the Bcl-2 19-kDa interacting protein 3 (BNIP3) can induce autophagy in many cancers^26^,^27^. BNIP3 is subjected to both proteasomal and autophagic degradation, and both degradation pathways of BNIP3 contribute to the different pathogenesis of many diseases^28^. In order to further clarify whether AhR-regulated autophagy is mediated by BNIP3 protein degradation, we confirmed the degradation pathway by treatment with the proteasome inhibitor MG132 (10 μM) and autophagy inhibitor BafA1 (5 nM). AhR-overexpressing CL1-5 cells showed a greater increase in BNIP3 accumulation following MG132 treatment compared to BafA1 treatment (Fig. 5b); a similar effect was observed in wt-A549 cells, which express high levels of AhR protein (Fig. 5a). Interestingly, wt-CL1-5 cells showed an inconsistent BNIP3 protein profile when compared to shAhR -A549 cells, particularly in the MG132-treated group. These results suggest that BNIP3 degradation through the autophagy or proteasome pathway further depends on AhR regulation, and strongly suggest that BNIP3 is an important indicator of the contribution of low AhR expression in increased autophagy.

### AhR decreased autophagy activation level by interacting with BNIP3 and via the ubiquitin-proteasome degradation pathway

**Figure 6:**
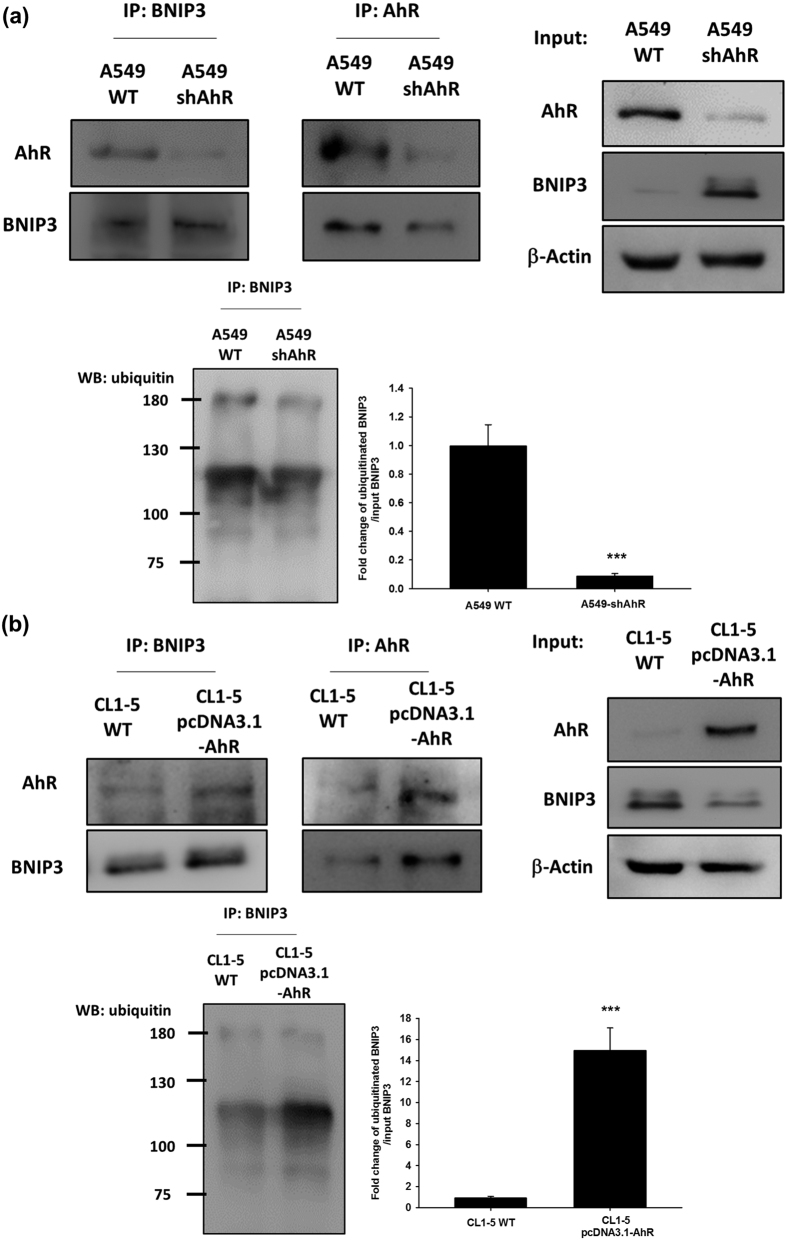
Protein-protein interaction between AhR, BNIP3, and ubiquitin was affected by AhR protein expression level. Immunocomplexes were captured with anti-AhR or anti-BNIP3 antibodies and then separated by SDS-PAGE. (**a**) The data showed that the AhR-BNIP3 interaction was decreased by AhR knockdown in A549 cells. Ubiquitination of BNIP3 also reduced with AhR silencing. (**b**) AhR-BNIP3 interaction was increased by AhR overexpression in CL1-5 cells. Ubiquitination of BNIP3 also increased with AhR overexpression. Full-length blots are presented in Supplementary Fig. S18. Ubiquitinated BNIP3 were normalized to total BNIP3 of input group. Quantified data were presented as means ± SD, ***P < 0.001 compared to the control group, which indicate a statistical difference between comparative groups.

Novel functions have recently been reported for AhR, and previous studies have indicated an atypical role for AhR in toxicology. Although the complicacy function of AhR remains unclear, it may be an E3 ubiquitin ligase independently of its transactivation function^29^. Herein, we investigated the degradation pathway of BNIP3 in an immunoprecipitation assay to evaluate the relationship between AhR and BNIP3 during autophagy in lung cancer. As shown in Fig. 6a, the protein complexes were captured using specific anti-AhR and anti-BNIP3 antibodies and both proteins were detected by immunoblotting. Compared to the control group (wt-A549), which clearly showed the presence of AhR/BNIP3 protein complexes, the shAhR group showed decreased levels of AhR/BNIP3 protein complexes. We also performed the assay using a non-denatured lysis sample (without 2-mercaptoethanol) of wt-A549 and AhR-silenced A549 cells to confirm the BNIP3/ubiquitin-conjugated super-complexes. Non-denatured lysis sample revealed ~120 kDa BNIP3 species probe with anti-BNIP3 which was used in co-IP of ubiquitinated assay (Fig. S7). AhR knockdown clearly decreased BNIP3 ubiquitination in A549 cells. Ubiquitinated BNIP3 was normalized to total BNIP3 of input panel on a histogram. Moreover, the results showed that AhR overexpression in CL1-5 cells enhanced the formation of AhR/BNIP3 protein complexes as demonstrated by capture with anti-AhR and anti-BNIP3 antibodies. Non-denatured lysis samples showed a dramatic increase in BNIP3 ubiquitination in AhR-overexpressing CL1-5 cells compared to in the wild type group (Fig. 6b). These results indicate that AhR plays a critical role in the ubiquitin-proteasome degradation pathway and strongly suggests that the AhR/BNIP3 protein-protein interaction contributes to cellular autophagy levels.

## Discussion

The estimated prognosis after diagnosis of late-stage lung cancer has been established as less than 1 year, with a high mortality rate because of cancer metastasis^30^,^31^. Over the last decade, several studies have found that autophagy is one of the most significant physiological events in the constitutive degradation of cellular proteins and is thus important for cell survival in different cancers. Interestingly, most recent studies have observed contradicting effects for autophagy, revealing both pro-metastatic and anti-metastatic roles in cancer metastasis^32^,^33^. In the present study, we found a novel crosstalk mechanism between cellular autophagy and AhR that is potentially involved in cellular EMT progression. We found that AhR protein expression was strongly associated with EMT and affects autophagy in NSCLC. AhR regulates autophagy and EMT through a pathway involving BNIP3 degradation. These results showed that AhR is a potential upstream therapeutic target for treating highly invasive NSCLC accompanied by autophagy.

AhR is a ligand-activated receptor that regulates xenobiotic metabolism. Some studies have suggested both tumour suppressive and oncogenic functions for ligand-activated AhR^10^; however, the other physiological functions of AhR remain unknown. The contradictory observations in AhR-modulated EMT in the context of cancer have not been clarified, particularly for 2,3,7,8-tetrachlorodibenzo-*p*-dioxin, which is a known human carcinogen as classified by the International Agency for Research on Cancer. The anti-metastatic role of 2,3,7,8-tetrachlorodibenzo-*p*-dioxin was suggested to involve AhR activation and decreased cancer cell survival in breast cancer^9^,^34^. Oestrogen-positive breast cancer shows a strong association between AhR regulation and cancer metastasis^35^. In contrast, AhR activation may augment cell migration through the Jun N-terminal kinase-dependent pathway, accompanied by nuclear localization of AhR in multiple cell culture models^36^. Few studies have examined AhR-modulated EMT without activation by extrinsic factors, but the exact mechanisms underlying the relationship between basal AhR levels and cellular EMT remain unknown. We found that a lower basal level of AhR was associated with high cell motility in lung cancer cells (Fig. 1). We also examined the localization of overexpressed AhR and found that the AhR protein was mainly localized to the cytosol without transactivation activity until ligand stimulation (Fig. S9). In addition, AhR overexpression or knockdown alone altered cellular EMT marker expression (Fig. 1). These findings indicate that AhR is not only activated by extrinsic factors, but also may have other endogenous functions in the cytosol for modulating EMT. Although endogenous ligands suggest a new axis for determining AhR function, our ongoing research is focused on whether other non-transactivation functions of AhR are involved in cellular EMT.

The mechanisms governing enhanced tumour cell EMT under conditions of low AhR expression are unclear. As described in this study, autophagy marker levels were altered and accompanied by different levels of AhR protein expression (Figs 2–4). Because autophagy maintains cellular homeostasis, cancer cells also use autophagy for survival^37^. A recent study reported that the autophagy strongly affects EMT progression; in contrast, EMT induced by TGF-β also affects autophagy flux, indicating that these processes regulate each other^38^. However, the mechanism by which autophagy directly modulates cancer cell invasion is unclear. In this study, we used a late-stage autophagy inhibitor, BafA1, rather than 3-MA (early-stage autophagy inhibitor) to confirm that autophagy inhibition was related to AhR function in lung cell EMT. 3-MA is an inhibitor of phosphatidylinositol 3-kinases; several studies have revealed that inhibition of the phosphatidylinositol 3-kinases/AKT pathway suppresses cell migration and invasion. Consistent with the results of previous studies^18^,^39^, although BafA1 treatment downregulated lung cancer cell motility (Fig. 2b,c), ATG12 siRNA and BNIP3 shRNA also impaired cell migration in CL1-5 cells (Fig. S2). In addition, the effects of cell proliferation and apoptosis have been related to cell migration^40^, we have also conducted the experiments and found that a decrease of cell proliferation in AhR overexpressing cells (Fig. S3a); and the apoptosis markers, especially BCL-2, and cleaveged-caspase3 protein expression were changed (data not shown). It is possible that the effect of AhR-regulated autophagy, which caused cell proliferation and apoptosis changed in cell migration. As these results above, which suggest that the cell may using autophagy to promote invasion indirectly, but not directly. On the other hand, our finding is consistent with previous studies, BNIP3 is required to maintain steady-state levels of intracellular complexes orchestrating the plasticity of the actin cytoskeleton, knockdown of BNIP3 could enhance melanoma cell migration^41^. However, the indirectly cause of invasive reduction by autophagy inhibition might be an effect in cell EMT^42^,^43^, thus knockdown of other autophagy-specific genes is required to further clarify this observation. Cadherin-switch is a known process in tumour progression. We found E-cadherin was downregulated while AhR knockdown in A549 cells. Low AhR expressed cells, H1299 and CL1-5 both within no E-cadherin expression. The interesting observation is AhR overexpression could not rescue E-cadherin expression both in H1299 and CL1-5 cells (Fig. S4). These results suggested that AhR may not a directly modulator of E-cadherin, so that E-cadherin levels expression was not rescued within AhR overexpression cells. Another possibility was indicated in a study, E-cadherin could be de-regulation by src activation^44^, but src activation could be an AhR-dependent or –independent pathway^45^,^46^. We also performed a pulmonary metastasis assay using a xenograft model *in vivo*. Both AhR-overexpression and BafA1 treatment abolished the metastatic foci in murine lungs (Fig. 2e). However, the results we have found *in vivo* with A549 cells were not fully consistent with the expectation in contrast to CL1-5 cells. Just a small metastatic tumor clone was observed, we think there are two possibilities in this case. First, in our study, we found large amounts of AhR presented in A549 rather than CL1-5 cell line. We found the results of cell invasion assay in Fig. 1F, which present similar invasive cells/field in AhR-silenced A549 and AhR overexpressing CL1-5 cells. Furthermore, *in vivo* results showed no tumour colonies in AhR overexpressing CL1-5 cells. These points indicated that CL1-5 cells reveals much more sensitive than A549 cells when changing AhR levels. Secondly, some studies have demonstrated that A549 cells with different metastatic potentials *in vivo*. The metastatic potential of A549 cells to the lungs were found approximately 7%^47^. Moreover, c-MET protein activation or not in A549 cells could contribute to different metastatic pattern among different organs. In this study, our results suggest that AhR is essential for regulating autophagy and further contributes to cellular EMT progression.

AhR-regulated autophagy is an important finding of this study, and the detailed mechanism depends on the BNIP3/AhR protein interaction (Fig. 6). AhR activation acts as a component of the E3 ubiquitin ligase complex; however, its exact role in this complex has not been established^48^. BNIP3 is involved in cell death and cell survival via autophagy^49^. Degradation of BNIP3 may directly affect autophagy flux. We found that a high level of AhR protein expression was associated with low autophagy activity (Figs 2d and 3), and further investigated BNIP3 degradation using the inhibitors MG132 and BafA1 (Fig. 5). However, there was no statistically difference between MG132 treatment, BafA1 treatment, and control groups in shAhR-A549 cells. There are two possibilities that may explain why BNIP3 accumulation in shAhR-A549 cells differed from that in wt-CL1-5 cells. First, the target shRNA we used to evaluate AhR knockdown in the A549 model was not sufficient for eliminating AhR function, suggesting that some posttranslational modifications remain to be identified. The second is that BNIP3-dependent autophagy in A549 cells is not only AhR-regulated, but is affected by other factors. The generation and investigation of complete *Ahr*-knockout mice would help further clarify this issue and reveal the biological significance of AhR-regulated autophagy. In this study, we found that higher AhR protein expression contributed to BNIP3 accumulation following MG132 treatment. The immunoprecipitation assay showed increased levels of the BNIP3/AhR complex in wt-A549 and AhR-overexpressing CL1-5 cells; the BNIP3-ubiquitin super complex was also enhanced in the presence of AhR protein (Fig. 6). In addition, BNIP3 was shown to be upregulated in such malignant tumours via HIF-α-mediated signalling in a hypoxic microenvironment^50^. Increasing evidence has also suggested that BNIP3 triggers autophagy by inducing mitophagy of defective mitochondria^51^,^52^. In the present study, we found that BNIP3 degradation is based on AhR expression, but not on activation by extrinsic factors. Although defective proteins that are rapidly recycled from the autolysosome in the autophagy process may contribute to cell migration in some cancers^53^, the role of the extent of autophagy in cellular EMT during cancer progression remains unclear, and analysis of BNIP3-dependent mitophagy based on AhR protein levels in NSCLC is warranted. Taken together, we found that AhR regulates BNIP3 degradation to disturb the autophagy process in lung cancer cells, which may result in decreased cellular EMT.

In summary, this is the first study of AhR-regulated autophagy in lung cancer EMT, which is dependent on the role of cellular autophagy in cancer metastasis and endogenous AhR functions. Although the regulatory crosstalk and interference in the endogenous AhR-autophagy-EMT signalling node is complicated, our study highlights the specific role of BNIP3 in AhR-regulated autophagy without activation by extrinsic factors, revealed the mechanism of this crosstalk, and reflected the basal function and potential of autophagy in the presence of AhR protein with respect to physiological implications related to NSCLC.

## Materials and Methods

### Chemicals

The autophagy inhibitor bafilomycin A1 and proteasome inhibitor MG132 were purchased from Cayman Chemical Company (88899-55-2 and 133407-82-6, respectively; Ann Arbor, MI, USA).

### Cell culture and treatment

A549 and H1299 cells were obtained from the American Type Culture Collection (Manassas, VA, USA). The CL1-5 cell lines were kindly provided by Prof. Chen Huei-Wen (Institute of Toxicology, National Taiwan University, Taipei, Taiwan). A549 and H1299 cells were maintained in Dulbecco’s modified Eagle’s medium (DMEM, 11995-123; Gibco, Grand Island, NY, USA); CL1-5 were maintained in Roswell Park Memorial Institute 1640 medium (31800-089; Gibco). Both media were supplemented with 10% heat-inactivated foetal bovine serum (Biological Industries, Kibbutz Beit-Haemek, Israel), 2 mM l-glutamine (ThermoFisher, Waltham, MA, USA), 10,000 U/mL penicillin, 10 mg/mL streptomycin, and 25 μg/mL amphotericin B (Biological Industries) incubated in a humidified atmosphere of 5% CO_2_ at 37 °C. Before testing, the cells were plated into 6-well plates for western blotting and mRNA analysis at a density of 2 × 10^5^ cells/mL for overnight incubation. The cells then were treated with BafA1 or MG132 for 24 h.

### Lentiviral transduction for AhR gene silencing and transfection for AhR overexpression

The VSV-G expression vector pMDG, gag-pol expression vector pCMVD8.91, and pLKO.1 encoding the AhR small hairpin RNA (shRNA) (TRCN0000245286; RNAi core, Taipei, Taiwan) with the target sequence 5′-CCGGCCCACAACAATATAATGTC TTCTCGAGAAGACATTATATTGTTGTGGGTTTTT-3′, were co-transfected into 293 T cells by calcium phosphate precipitation. All plasmids were obtained from the National RNAi core facility (Academic Sinica, Taiwan). The virus-containing supernatants were collected after 24–48 h of transfection and were filtered through a 0.45-μm syringe filter. The viral supernatants were added to the A549 culture in the presence of 10 μg/mL polybrene (hexadimethrine bromide; 107689; Sigma, St. Louis, MO, USA). After 72 h of infection, lentivirus-infected A549 cells were used subsequent studies. Overexpression of human AhR was achieved by transfection with pcDNA3.1-AhR, which contained the full-length AhR gene (GenBank Accession number 196, NCBI) and was constructed using the pcDNA™3.1/V5-His TOPO^®^ TA expression kit (Invitrogen, Carlsbad, CA, USA) according to the manufacturer’s protocols. Transient transfection of cells was performed using Turbofect™ (Thermo Fisher) according to the manufacturer’s instructions to obtain AhR-overexpressing cells.

### Western blotting and antibodies

Whole-cell lysate in radioimmunoprecipitation assay buffer, determination of protein concentration, SDS-PAGE, and immunoblotting were performed as described previously^54^. For immunodetection, polyvinylidene fluoride membranes were blocked with 5% non-fat milk, incubated in Tris-buffered saline (pH 7.6; Sigma). The membranes were then incubated overnight at 4 °C with specific antibodies as follows: mouse monoclonal anti-AhR (sc-74571; Santa Cruz Biotechnology, Santa Cruz, CA, USA) and anti-p62/SQSTM1 (sc-28359; Santa Cruz Biotechnology); rabbit monoclonal anti-LC3B (GTX127375; Genetex, Irvine, CA, USA), anti-ATG12-5 (GTX124181; Genetex), anti-ATG7 (GTX61647; Genetex), and anti-BNIP3 (ab109362; Abcam, Cambridge, UK), rabbit polyclonal anti-E-cadherin (GTX100443; Genetex) anti-vimentin (GTX100619; Genetex); and mouse monoclonal anti-β-actin (A1978; Sigma). The blots were then incubated with HRP-conjugated anti-rabbit and anti-mouse IgG antibodies obtained from Cell Signaling Technology (7074 and 7076; Danvers, MA, USA). Enhanced chemiluminescence detection was performed according to the manufacturer’s protocol (Millipore, Billerica, MA, USA).

### Transwell cell migration and invasion assay

The cell migration and invasion assays were conducted as described previously^55^. Briefly, after transfection of shAhR and pcDNA3.1-AhR for 72 h and 24 h, respectively, Transwells were used for these assays. First, 2 × 10^5^ cells (upper chamber with no matrigel coating for migration assay) and 10^6^ cells per well were seeded into the upper invasion chamber (8 μm) which was coated Matrigel (for invasion assay) and placed in 24-well plates. Next, 10% FBS in DMEM was added to the lower invasion chamber. After 16 h incubation, the Transwell insert was removed from the plate. A cotton-tipped applicator was used to carefully remove the remaining cells that had not migrated/invaded from the top of the membrane. The membrane was washed twice with PBS, fixed with 3.7% formaldehyde for 15 min, and stained with crystal violet. The cells were observed and photographed under a light microscope.

### Immunoprecipitation analysis

Whole cell lysates were pre-cleared by short incubation with protein A magnetic beads (Millipore). The lysates (1 mg) were then subjected to immunoprecipitation by the addition of 1 μg/mL anti-AhR and anti-BNIP3 antibodies, and incubated overnight with rotation at 4 °C. This was followed by shaking with 50 μL protein A magnetic beads for an additional 2 h. The captured immunocomplexes were precipitated and washed three times with radioimmunoprecipitation assay buffer prior to addition of 100 μL of 2 × SDS sample buffer and non-denaturing lysis sample in 2 × SDS sample buffer without 2-mercaptoethanol. Samples were heated at 95 °C for 6 min, electrophoresed on 7.5% SDS–polyacrylamide gels, and analysed by immunoblotting as described above.

### Transmission electron microscopy (TEM)

After different treatments as indicated, the cells were collected and washed with PBS then fixed in 2.5% glutaraldehyde overnight. Then, the cells washed by 0.1 M PBS and fixed with 1% OsO4. Next, the cells were dehydrated using a range of alcohol concentrations for 15 minutes. The cells were then embedded into paraffin and sliced using an LKB-V ultramicrotome (BROMMA, Sweden). For TEM images acquisition, the prepared sections were examined on a JEM-2100 microscope operating at 120 kV (JEOL Ltd., Tokyo, Japan).

### Confocal immunofluorescence assay

A549 (vector control and shA groups) and CL1-5 cells (vector control and AhR overexpression groups) were seeded in 35-mm dishes containing coverslips and incubated for 16 h. Cell were fixed in 4% paraformaldehyde with PBS for 15 min, followed by treatment with 0.1% Triton–X100 for 5 min. Next, 2% BSA was used as blocking buffer and incubated with the cells for 30 min. Primary antibodies of LC3B antibody were diluted to their final concentration in 2% BSA and incubated for 1 h with a coverslip. Diluted secondary antibody Alexa Fluor 488 (Jackson ImmunoResearch, West Grove, PA, USA) was added for 30 min after which nuclei were stained with Hoechst 33258 (Sigma) for 5 min. Fluorescence images were then observed under the Leica TCS SP5 confocal microscopy system (Leica, Wetzlar, Germany).

### Real-time transcription-polymerase chain reaction (real-time RT-PCR)

RNA samples were prepared using the TriPure reagent according to the manufacturer’s protocol (Roche, Basel, Switzerland). BNIP3 levels were analysed by real time RT-PCR and normalized to that of the housekeeping gene, β-actin, as an internal control. Briefly, first-strand cDNA was synthesised from 6 μg of total RNA at 37 °C for 60 min using MMLV high-performance reverse transcriptase (MM070150; Epicentre, Madison, WI, USA). For real-time PCR, a Roche LightCycler Nano (Roche) and iQ™ SYBR^®^ Green Supermix (Bio-Rad Laboratories, Inc., Hercules, CA, USA) were used. cDNA was amplified using specific primers. The primer sets were as follows: AhR (sense, 5′-GGCTTGGAATTACAGGAATCC-3′, antisense, 5′-CAGCCTCAGGATGTGAACTC-3′), BNIP3 (sense, 5′-GCTCCCAGACACCACAAGAT-3′, antisense, 5′-TGAGAGTAGCTGTGCGCTTC-3′), LC3 (sense, 5′-ATGCCGTCGGAGAAGAC CTT-3′, antisense, 5′-TTACACTGACAATTTCATCCCG-3′), GADPH (sense, 5′-ACCCA GAAGACTGTGGATGG-3′, antisense, 5′-CAGTGAGCTTCCCGTTCAG-3′) on a DNA thermal cycler (Applied Biosystems, Foster City, CA, USA) using the following program: denaturation for 5 min at 95 °C, followed by 35 cycles of denaturation for 30 s at 95 °C, annealing for 30 s at 55 °C, and extension for 40 s at 72 °C, with a final extension for 10 min at 72 °C. The PCR products were separated by 1.5% agarose gel electrophoresis and visualized by ethidium bromide staining.

### Pulmonary metastasis assay

Ethical Guidelines for present study was approved by the Animal Care Committee of the National Taiwan University, College of Medicine (IACUC’s number: 20130450), The protocol for developing the mouse xenograft model was approved by the Experimental Animal Center of National Taiwan University, College of Medicine. The Committee recognizes that the proposed animal experiment follows the Animal Protection Law by the Council of Agriculture, Executive Yuan, Taiwan. Animals were handled in all experiments in accordance with the Guide for the Care and Use of Laboratory Animals (National Academy of Sciences Press, 1996). The *in vivo* metastatic potential and tumorigenic abilities of wt-A549, shAhR-A549, wt-CL1-5, and AhR-overexpressing CL1-5 cells were measured using lung colonization in a xenograft model^56^. ICR mice were obtained from the National Taiwan University Animal Center and housed aseptically in its animal facilities. The animals were randomly divided into experimental groups, and the groups were treated as follows: For the lung colonization assay, a single-cell suspension (1 × 10^6^ cells) of wt-A549, shAhR-A549, wt-CL1-5, and AhR-overexpressing CL1-5 cells was prepared in 0.1 mL serum-free DMEM and then injected into the tail vein of 8-week-old ICR mice. BafA1 was given to mice by i.p. injection (0.3 mg/kg/day) After 40 days, the mice were anesthetised with isoflurane and sacrificed. The metastatic colonies on the lung surface were observed.

### Haematoxylin eosin (HE) staining and immunohistochemistry

Lung specimens from mice were dehydrated in ethanol and embedded in paraffin. Radial 5-μm sections were collected for haematoxylin and eosin (HE) staining. For immunohistochemistry, lung specimens were fixed in 10% formalin and subsequently dehydrated, paraffin-embedded, and sectioned. Lung specimens were subjected to antigen retrieval with microwave irradiation in a citrate buffer (10 mM, pH 6.0) for 10 min. The sections were incubated at 4 °C with primary antibody overnight. Anti-human BNIP3 (1:500) was used for immunohistochemistry. For immunohistochemical staining, the sections were incubated with corresponding HRP-conjugated secondary antibodies at room temperature for 1 h and visualized using 0.05% 3, 3′-diaminobenzidine, and the nuclei were counterstained with haematoxylin.

### Statistical analysis

All data are expressed as the mean ± standard deviation (SD) from at least three independent experiments (n ≥ 3). Statistically significant differences between the control and each experimental condition were analysed using the Student’s *t*-test. Statistically significant differences among groups were determined by one-way analysis of variance. P < 0.05 was considered as statistically significant.

## Additional Information

**How to cite this article**: Tsai, C.-H. *et al*. The inhibition of lung cancer cell migration by AhR-regulated autophagy. *Sci. Rep.* **7**, 41927; doi: 10.1038/srep41927 (2017).

**Publisher's note:** Springer Nature remains neutral with regard to jurisdictional claims in published maps and institutional affiliations.

## Supplementary Material

Supplementary Information

## References

[b1] Siegel R. L., Miller K. D. & Jemal A. Cancer statistics, 2015. CA Cancer J Clin 65, 5–29, doi: 10.3322/caac.21254 (2015). 25559415

[b2] Scheff R. J. & Schneider B. J. Non-small-cell lung cancer: treatment of late stage disease: chemotherapeutics and new frontiers. Semin Intervent Radiol 30, 191–198, doi: 10.1055/s-0033-1342961 (2013). 24436536 PMC3710022

[b3] Wong I. C., Ng Y. K. & Lui V. W. Cancers of the lung, head and neck on the rise: perspectives on the genotoxicity of air pollution. Chin J Cancer 33, 476–480, doi: 10.5732/cjc.014.10093 (2014). 25011457 PMC4198750

[b4] Krishnan V. G. . Whole-genome sequencing of asian lung cancers: second-hand smoke unlikely to be responsible for higher incidence of lung cancer among Asian never-smokers. Cancer Res 74, 6071–6081, doi: 10.1158/0008-5472.CAN-13-3195 (2014). 25189529

[b5] Rubin H. Synergistic mechanisms in carcinogenesis by polycyclic aromatic hydrocarbons and by tobacco smoke: a bio-historical perspective with updates. Carcinogenesis 22, 1903–1930 (2001). 11751421 10.1093/carcin/22.12.1903

[b6] Gu Y. Z., Hogenesch J. B. & Bradfield C. A. The PAS superfamily: sensors of environmental and developmental signals. Annu Rev Pharmacol Toxicol 40, 519–561, doi: 10.1146/annurev.pharmtox.40.1.519 (2000). 10836146

[b7] Bersten D. C., Sullivan A. E., Peet D. J. & Whitelaw M. L. bHLH-PAS proteins in cancer. Nat Rev Cancer 13, 827–841, doi: 10.1038/nrc3621 (2013). 24263188

[b8] Nebert D. W., Dalton T. P., Okey A. B. & Gonzalez F. J. Role of aryl hydrocarbon receptor-mediated induction of the CYP1 enzymes in environmental toxicity and cancer. J Biol Chem 279, 23847–23850, doi: 10.1074/jbc.R400004200 (2004). 15028720

[b9] Safe S., Lee S. O. & Jin U. H. Role of the aryl hydrocarbon receptor in carcinogenesis and potential as a drug target. Toxicol Sci 135, 1–16, doi: 10.1093/toxsci/kft128 (2013). 23771949 PMC3748760

[b10] Murray I. A., Patterson A. D. & Perdew G. H. Aryl hydrocarbon receptor ligands in cancer: friend and foe. Nat Rev Cancer 14, 801–814, doi: 10.1038/nrc3846 (2014). 25568920 PMC4401080

[b11] Thiery J. P. Epithelial-mesenchymal transitions in tumour progression. Nat Rev Cancer 2, 442–454, doi: 10.1038/nrc822 (2002). 12189386

[b12] Tester A. M., Ruangpanit N., Anderson R. L. & Thompson E. W. MMP-9 secretion and MMP-2 activation distinguish invasive and metastatic sublines of a mouse mammary carcinoma system showing epithelial-mesenchymal transition traits. Clin Exp Metastasis 18, 553–560 (2000). 11688960 10.1023/a:1011953118186

[b13] Yuki K., Yoshida Y., Inagaki R., Hiai H. & Noda M. E-cadherin-downregulation and RECK-upregulation are coupled in the non-malignant epithelial cell line MCF10A but not in multiple carcinoma-derived cell lines. Sci Rep 4, 4568, doi: 10.1038/srep04568 (2014). 24691523 PMC3972504

[b14] Zeisberg M. & Neilson E. G. Biomarkers for epithelial-mesenchymal transitions. J Clin Invest 119, 1429–1437, doi: 10.1172/JCI36183 (2009). 19487819 PMC2689132

[b15] Mendez M. G., Kojima S. & Goldman R. D. Vimentin induces changes in cell shape, motility, and adhesion during the epithelial to mesenchymal transition. FASEB J 24, 1838–1851, doi: 10.1096/fj.09-151639 (2010). 20097873 PMC2874471

[b16] White E. Deconvoluting the context-dependent role for autophagy in cancer. Nat Rev Cancer 12, 401–410, doi: 10.1038/nrc3262 (2012). 22534666 PMC3664381

[b17] Lavieu G. . Is autophagy the key mechanism by which the sphingolipid rheostat controls the cell fate decision? Autophagy 3, 45–47 (2007). 17035732 10.4161/auto.3416

[b18] Li J. . Autophagy promotes hepatocellular carcinoma cell invasion through activation of epithelial-mesenchymal transition. Carcinogenesis 34, 1343–1351, doi: 10.1093/carcin/bgt063 (2013). 23430956

[b19] Portal-Nunez S. . Aryl hydrocarbon receptor-induced adrenomedullin mediates cigarette smoke carcinogenicity in humans and mice. Cancer Res 72, 5790–5800, doi: 10.1158/0008-5472.CAN-12-0818 (2012). 22993405 PMC4340077

[b20] Gluschnaider U. . beta-TrCP inhibition reduces prostate cancer cell growth via upregulation of the aryl hydrocarbon receptor. PLoS One 5, e9060, doi: 10.1371/journal.pone.0009060 (2010). 20140206 PMC2816705

[b21] Stricker J., Falzone T. & Gardel M. L. Mechanics of the F-actin cytoskeleton. J Biomech 43, 9–14, doi: 10.1016/j.jbiomech.2009.09.003 (2010). 19913792 PMC2813332

[b22] Morris H. T. & Machesky L. M. Actin cytoskeletal control during epithelial to mesenchymal transition: focus on the pancreas and intestinal tract. Br J Cancer 112, 613–620, doi: 10.1038/bjc.2014.658 (2015). 25611303 PMC4333498

[b23] Choi K. S. Autophagy and cancer. Exp Mol Med 44, 109–120, doi: 10.3858/emm.2012.44.2.033 (2012). 22257886 PMC3296807

[b24] Akalay I. . EMT impairs breast carcinoma cell susceptibility to CTL-mediated lysis through autophagy induction. Autophagy 9, 1104–1106, doi: 10.4161/auto.24728 (2013). 23635487 PMC3722321

[b25] Klionsky D. J. . Guidelines for the use and interpretation of assays for monitoring autophagy (3rd edition). Autophagy 12, 1–222, doi: 10.1080/15548627.2015.1100356 (2016). 26799652 PMC4835977

[b26] Hamacher-Brady A. . Response to myocardial ischemia/reperfusion injury involves Bnip3 and autophagy. Cell Death Differ 14, 146–157, doi: 10.1038/sj.cdd.4401936 (2007). 16645637

[b27] Azad M. B. . Hypoxia induces autophagic cell death in apoptosis-competent cells through a mechanism involving BNIP3. Autophagy 4, 195–204 (2008). 18059169 10.4161/auto.5278 PMC3164855

[b28] Park C. W. . BNIP3 is degraded by ULK1-dependent autophagy via MTORC1 and AMPK. Autophagy 9, 345–360, doi: 10.4161/auto.23072 (2013). 23291726 PMC3590255

[b29] Ohtake F. . Dioxin receptor is a ligand-dependent E3 ubiquitin ligase. Nature 446, 562–566, doi: 10.1038/nature05683 (2007). 17392787

[b30] Lutz S. . Symptom frequency and severity in patients with metastatic or locally recurrent lung cancer: a prospective study using the Lung Cancer Symptom Scale in a community hospital. J Palliat Med 4, 157–165, doi: 10.1089/109662101750290191 (2001). 11441624

[b31] Temel J. S. . Early palliative care for patients with metastatic non-small-cell lung cancer. N Engl J Med 363, 733–742, doi: 10.1056/NEJMoa1000678 (2010). 20818875

[b32] Su Z., Yang Z., Xu Y., Chen Y. & Yu Q. Apoptosis, autophagy, necroptosis, and cancer metastasis. Mol Cancer 14, 48, doi: 10.1186/s12943-015-0321-5 (2015). 25743109 PMC4343053

[b33] Kenific C. M., Thorburn A. & Debnath J. Autophagy and metastasis: another double-edged sword. Curr Opin Cell Biol 22, 241–245, doi: 10.1016/j.ceb.2009.10.008 (2010). 19945838 PMC2854304

[b34] Hsu E. L. . A proposed mechanism for the protective effect of dioxin against breast cancer. Toxicol Sci 98, 436–444, doi: 10.1093/toxsci/kfm125 (2007). 17517823

[b35] Jin U. H., Lee S. O., Pfent C. & Safe S. The aryl hydrocarbon receptor ligand omeprazole inhibits breast cancer cell invasion and metastasis. BMC Cancer 14, 498, doi: 10.1186/1471-2407-14-498 (2014). 25011475 PMC4226953

[b36] Diry M. . Activation of the dioxin/aryl hydrocarbon receptor (AhR) modulates cell plasticity through a JNK-dependent mechanism. Oncogene 25, 5570–5574, doi: 10.1038/sj.onc.1209553 (2006). 16619036

[b37] Lee Y. J. & Jang B. K. The Role of Autophagy in Hepatocellular Carcinoma. Int J Mol Sci 16, 26629–26643, doi: 10.3390/ijms161125984 (2015). 26561802 PMC4661843

[b38] Grassi G. . Autophagy regulates hepatocyte identity and epithelial-to-mesenchymal and mesenchymal-to-epithelial transitions promoting Snail degradation. Cell Death Dis 6, e1880, doi: 10.1038/cddis.2015.249 (2015). 26355343 PMC4650445

[b39] Macintosh R. L. . Inhibition of autophagy impairs tumor cell invasion in an organotypic model. Cell Cycle 11, 2022–2029, doi: 10.4161/cc.20424 (2012). 22580450 PMC3359125

[b40] Shang N. . FAK deletion accelerates liver regeneration after two-thirds partial hepatectomy. Sci Rep 6, 34316, doi: 10.1038/srep34316 (2016). 27677358 PMC5039626

[b41] Maes H. . BNIP3 supports melanoma cell migration and vasculogenic mimicry by orchestrating the actin cytoskeleton. Cell Death Dis 5, e1127, doi: 10.1038/cddis.2014.94 (2014). 24625986 PMC3973222

[b42] Niu N. K. . Pro-apoptotic and pro-autophagic effects of the Aurora kinase A inhibitor alisertib (MLN8237) on human osteosarcoma U-2 OS and MG-63 cells through the activation of mitochondria-mediated pathway and inhibition of p38 MAPK/PI3K/Akt/mTOR signaling pathway. Drug Des Devel Ther 9, 1555–1584, doi: 10.2147/DDDT.S74197 (2015). PMC4362906 25792811

[b43] Thorburn A., Thamm D. H. & Gustafson D. L. Autophagy and cancer therapy. Mol Pharmacol 85, 830–838, doi: 10.1124/mol.114.091850 (2014). 24574520 PMC4014668

[b44] Avizienyte E. . Src-induced de-regulation of E-cadherin in colon cancer cells requires integrin signalling. Nat Cell Biol 4, 632–638, doi: 10.1038/ncb829 (2002). 12134161

[b45] Frauenstein K. . Activation of the aryl hydrocarbon receptor by the widely used Src family kinase inhibitor 4-amino-5-(4-chlorophenyl)-7-(dimethylethyl)pyrazolo[3,4-d]pyrimidine (PP2). Arch Toxicol 89, 1329–1336, doi: 10.1007/s00204-014-1321-8 (2015). 25082669 PMC4454626

[b46] Tomkiewicz C. . The aryl hydrocarbon receptor regulates focal adhesion sites through a non-genomic FAK/Src pathway. Oncogene 32, 1811–1820, doi: 10.1038/onc.2012.197 (2013). 22665056

[b47] Jakubowska M. . Pulmonary metastases of the A549-derived lung adenocarcinoma tumors growing in nude mice. A multiple case study. Acta Biochim Pol 60, 323–330 (2013). 23828777

[b48] Ohtake F., Fujii-Kuriyama Y. & Kato S. AhR acts as an E3 ubiquitin ligase to modulate steroid receptor functions. Biochem Pharmacol 77, 474–484, doi: 10.1016/j.bcp.2008.08.034 (2009). 18838062

[b49] Tan E. Y. . BNIP3 as a progression marker in primary human breast cancer; opposing functions in *in situ* versus invasive cancer. Clin Cancer Res 13, 467–474, doi: 10.1158/1078-0432.CCR-06-1466 (2007). 17255267

[b50] Sowter H. M. . Expression of the cell death genes BNip3 and NIX in ductal carcinoma *in situ* of the breast; correlation of BNip3 levels with necrosis and grade. J Pathol 201, 573–580, doi: 10.1002/path.1486 (2003). 14648660

[b51] Chourasia A. H. . Mitophagy defects arising from BNip3 loss promote mammary tumor progression to metastasis. EMBO Rep 16, 1145–1163, doi: 10.15252/embr.201540759 (2015). 26232272 PMC4576983

[b52] Chourasia A. H. & Macleod K. F. Tumor suppressor functions of BNIP3 and mitophagy. Autophagy 11, 1937–1938, doi: 10.1080/15548627.2015.1085136 (2015). 26315353 PMC4824596

[b53] Perera R. M. . Transcriptional control of autophagy-lysosome function drives pancreatic cancer metabolism. Nature 524, 361–365, doi: 10.1038/nature14587 (2015). 26168401 PMC5086585

[b54] Tsai C. H. . NcoA2-Dependent Inhibition of HIF-1alpha Activation Is Regulated via AhR. Toxicol Sci 148, 517–530, doi: 10.1093/toxsci/kfv199 (2015). 26350169

[b55] Justus C. R., Leffler N., Ruiz-Echevarria M. & Yang L. V. *In vitro* cell migration and invasion assays. J Vis Exp, doi: 10.3791/51046 (2014). PMC4186330 24962652

[b56] Li C. H., Cheng Y. W., Liao P. L., Yang Y. T. & Kang J. J. Chloramphenicol causes mitochondrial stress, decreases ATP biosynthesis, induces matrix metalloproteinase-13 expression, and solid-tumor cell invasion. Toxicol Sci 116, 140–150, doi: 10.1093/toxsci/kfq085 (2010). 20338993 PMC2886854

