# Supplementary material for: The inhibition of lung cancer cell migration by AhR-regulated autophagy
Source: Sci Rep. 2017 Feb 14;7:41927. doi: 10.1038/srep41927 (PMC5307309; doi:10.1038/srep41927)
Supplement: Supplementary Information [file srep41927-s1.pdf]

Supplementary information

The inhibition of lung cancer cell migration by AhR-regulated autophagy

Chi-Hao Tsai[a], Ching-Hao Li[b], Yu-Wen Cheng[c], Chen-Chen Lee[d], Po-Lin Liao[a,c], Cheng-Hui Lin[c], Shih-Hsuan Huang[c], and Jaw-Jou Kang[a,*]

[a]Institute of Toxicology, College of Medicine, National Taiwan University, Taipei, Taiwan

[b]Department of Physiology, School of Medicine, College of Medicine, Taipei Medical University, Taipei, Taiwan

[c]School of Pharmacy, College of Pharmacy, Taipei Medicine University, Taipei, Taiwan

[d]Department of Microbiology and Immunology, School of Medicine, China Medicine University, Taichung, Taiwan

***Correspondence to:**

Jaw-Jou Kang, PhD

Institute of Toxicology, College of Medicine, National Taiwan University, Taipei, Taiwan

Tel: 886-2-23123456; Ext. 88603; Fax: 886-2-27374622

E-mail: jjkang@ntu.edu.tw

Target genes	Sequence 5’- 3’	Amplicon size (bp)
**AhR**	sense, 5′-GGCTTGGAATTACAGGAATCC-3′<br>antisense, 5′-CAGCCTCAGGATGTGAACTC-3′	1011
**Vimentin**	sense, 5′-GAGAACTTTGCCGTTGAAGC-3'<br>antisense, 5′-GCTTCCTGTAGGTGGCAATC-3′	163
**β-catenin**	sense, 5′-TTGATGGAGTTGGACATGG-3′<br>antisense, 5′-CAGGACTTGGGAGGTATCCA-3′	171
**Snail**	sense, 5′- ACTATGCCGCGCTCTTTCCT-3′<br>antisense, 5′- AGTCCTGTGGGGCTGATGTG-3′	943
**BNIP3**	sense, 5′-GCTCCCAGACACCACAAGAT-3′<br>antisense, 5′-TGAGAGTAGCTGTGCGCTTC-3′	225
**β-actin**	sense, 5′-TCATGAGGTAGTCAGTCAGG-3′<br>antisense, 5′-TGACCCAGATCATGTTTGAG-3′	215
**LC3**	sense, 5′-ATGCCGTCGGAGAAGACCTT-3′<br>antisense, 5′-TTACACTGACAATTTCATCCCG-3′	139
**GADPH**	sense, 5′-ACCCA GAAGACTGTGGATGG-3′<br>antisense, 5′-CAGTGAGCTTCCCGTTCAG-3′	139

Supplementary Table 1. The primer sets used in this study.

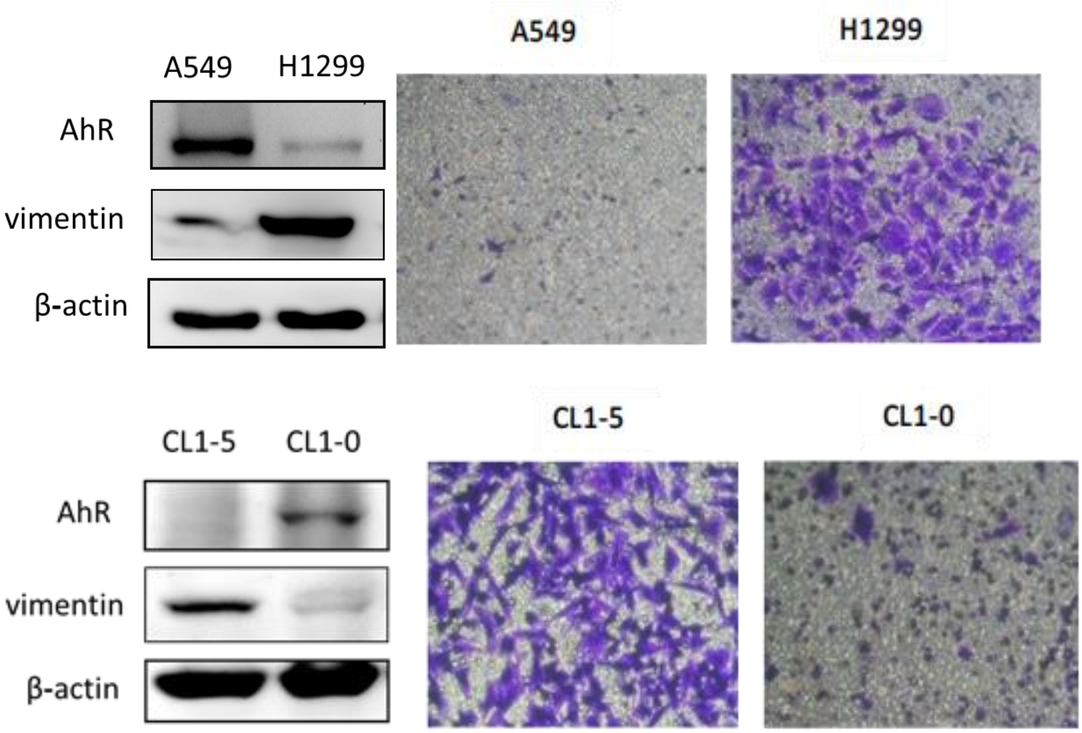

Supplementary Figure S1. The level of Aryl hydrocarbon receptor (AhR) expression in different cells was correlated with their vimentin expression and invasive potential. Cells (5x10^4 cells/transwell) were seeded on Matrigel- coated transwell inserts, incubated for 16 hours, and the invasive cells were stained and counted as described in the materials and methods section. AhR and vimentin expression was evaluated by western blotting. We found that cells that exhibited a higher invasive potential expressed lower levels of AhR. The images were acquired at 40X magnification.

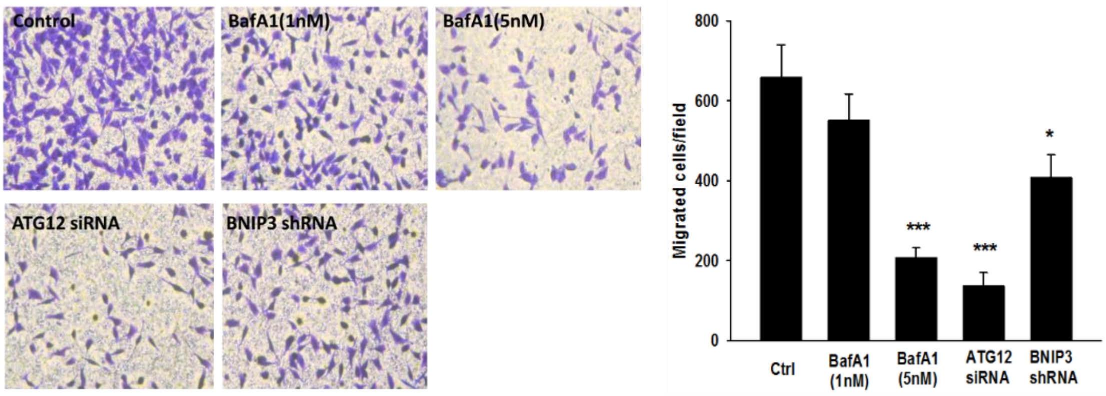

Supplementary Figure S2. Inhibition of autophagy impaired cell migration in CL1-5 cells. Cells were treated by BafA1 1nM, 5nM for 24h. Gene knockdown of ATG12 and BNIP3 were transfected with siRNA and shRNA for 72h, respectively. Cells($5x10^4$ cells/transwell) were seeded on Matrigel- coated transwell inserts, incubated for 16 hours, and the invasive cells were stained and counted as described in the materials and methods section. The quantified data were analysed and expressed as the mean ± SD from three independent experiments. **P < 0.01; ***P < 0.001 compared to the control group.

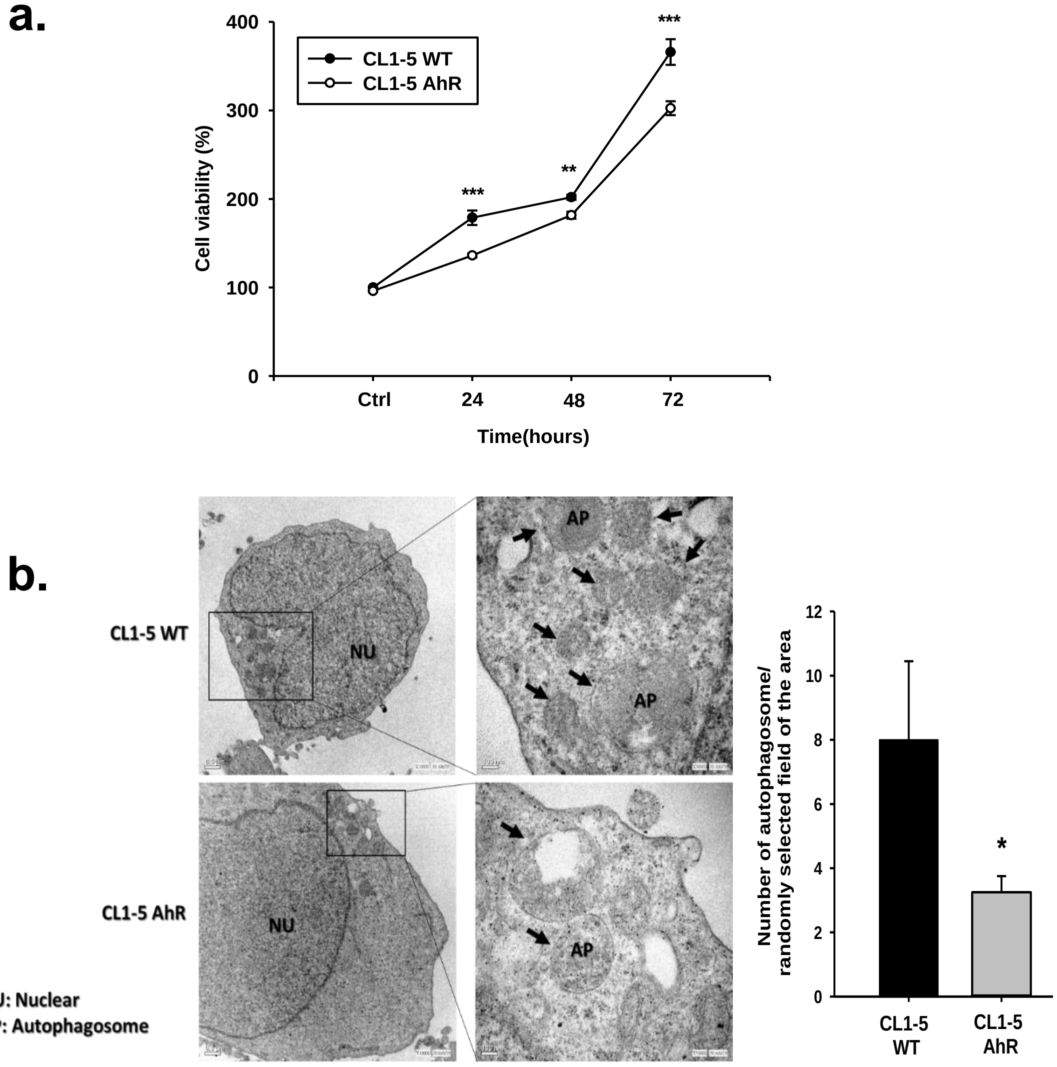

Supplementary Figure S3. The cell proliferation and TEM-depicted autophagosome ultrastructure in AhR-overexpressing CL1-5 cells. CL1-5 cells were transfection with pcDNA3.1-AhR for 24 h. (a) Cell proliferation of CL1-5 wt and CL1-5 AhR-overexpressed cell in 24, 48, 72h in culture. (b) Numerous autophagical vacuoles with typical double-layer membrane containing organelle remnants were highlighted by arrows. The images were acquired at 0.5μm and 100nm. The quantified data were analysed and expressed as the mean ± SD from

three independent experiments. *P < 0.05; **P < 0.01; ***P < 0.001compared to the control group.

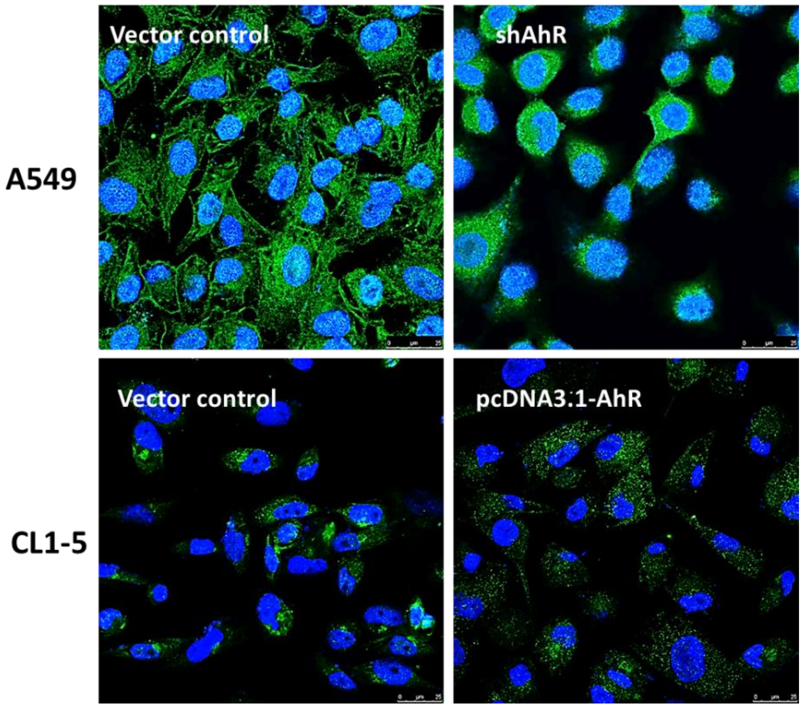

Supplementary Figure S4. AhR knockdown eliminated E-cadherin expression, but AhR overexpression could not rescue the expression of E-cadherin. The expression of E-cadherin was identified by immunofluorescence staining with FITC-conjugated antibodies and detected by Zeiss AXIO imager (40x). The nucleus was stained by Hoechst 33258.

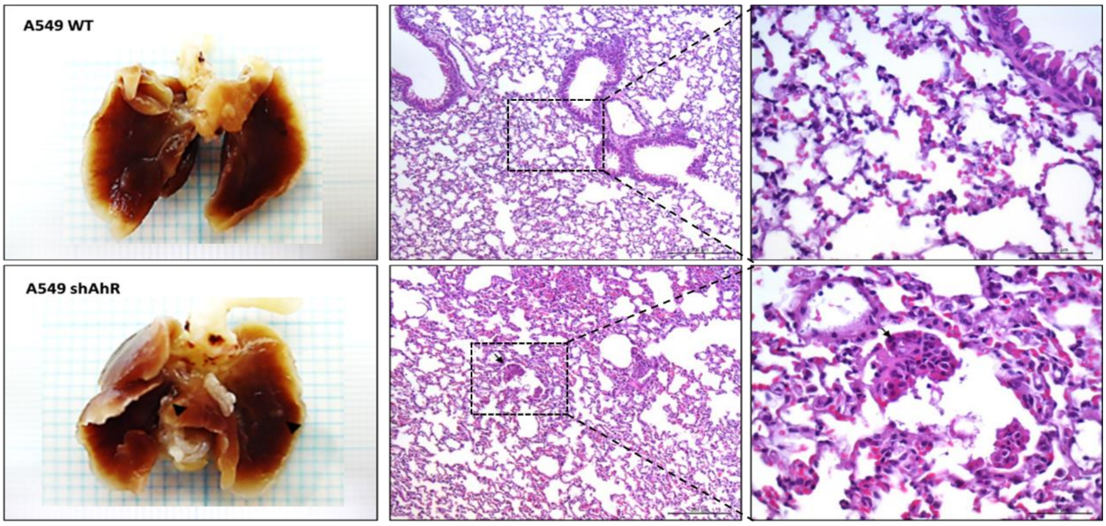

Supplementary Figure S5. A549 wt: No significant histopathological changes in the lung were observed. A549 shAhR: Small metastatic foci (arrow A) were detected in the lung. The metastatic tumor cells had small, round to oval, hyperchromatic nuclei, and abundant eosinophilic cytoplasm (A: scale bars measure 200 µm; B: scale bars measure 50 µm, H&E staining).

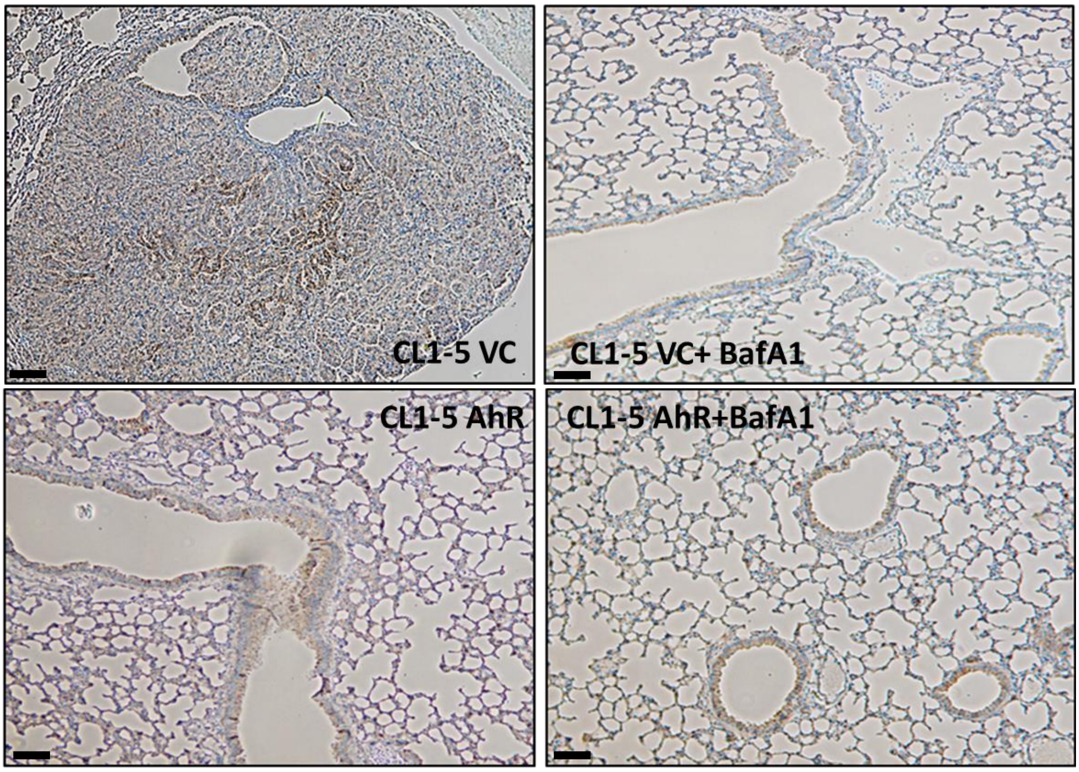

Supplementary Figure S6. Immunohistochemistry analysis revealed higher BNIP3 expression in the wt-CL1-5 group than in the other groups. The sample were collected from *in vivo* metastasis assay as performed on specimens using either anti-BNIP3. The anti-BNIP3 antibody was used (1:500) for immunohistochemistry.Positive cells were visualized by DAB staining. Scale bar, 100 μm.

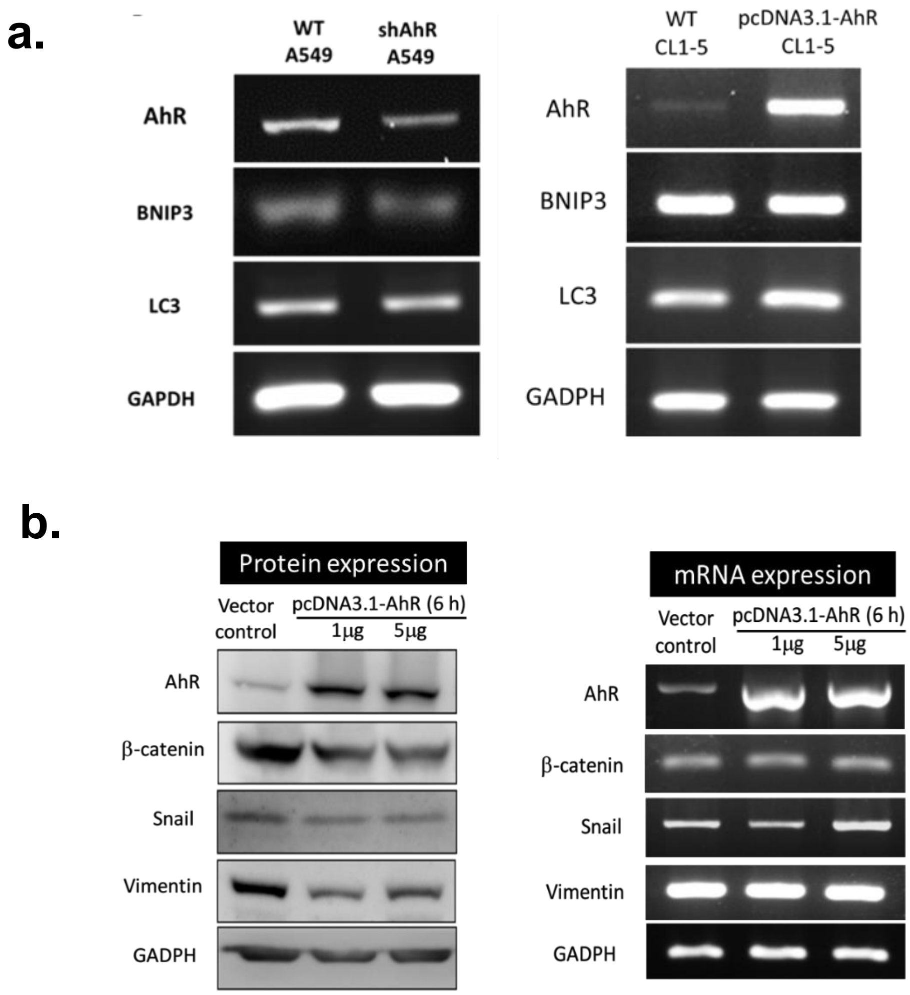

Supplementary Figure S7. AhR-knockdown or overexpression did not affect autophagy and EMT related gene expression in both A549 and CL1-5 cells. (a.) mRNA was isolated for RT-PCR after 48 hours of transduction and autophagy-related genes were then analyzed. BNIP3 and LC3 mRNA levels were unaffected by AhR changed in A549 and CL1-5 cells. (b) Protein level of EMT markers were screened by Western blots, only vimentin was definitely decreased in AhR-overexpressed CL1-5, whereas the expression of vimentin mRNA remain unaffected.

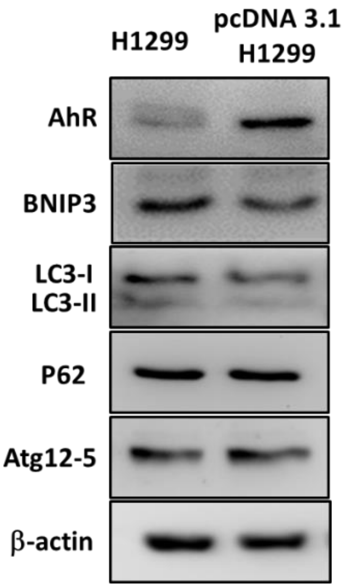

Supplementary Figure S8. AhR-overexpression decreased autophagy in H1299. AhR expression vector was transfected in H1299 cells using the Turbofect^{TM} transfection reagent for 24 hours, after which proteins were isolated for western blotting to analyze autophagy -related proteins. Western blot showed that AhR overexpression decreased BNIP3 and LC3 protein expression in H1299 cells.

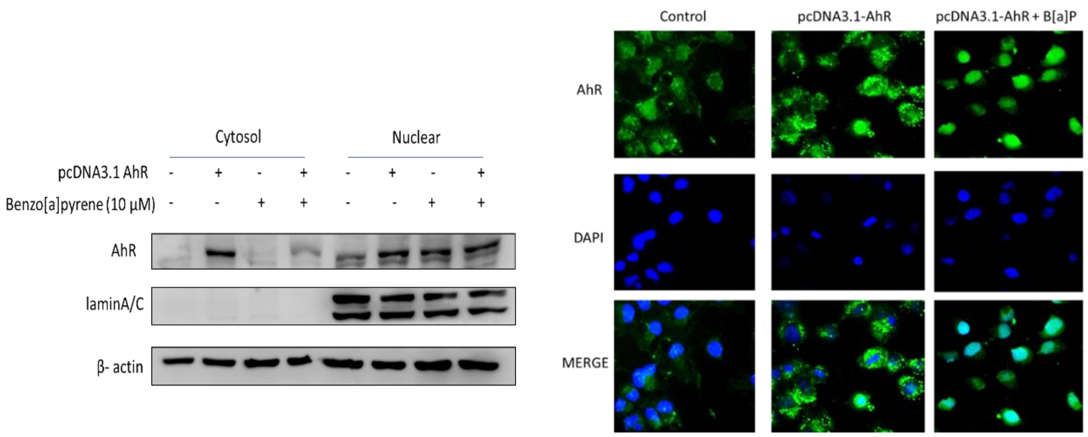

Supplementary Figure S9.Overexpressed AhR protein was mainly restricted in cytosol without transactivation activity until ligand stimulation. The cytosolic location of overexpressed AhR was confirmed by nuclear fractionation. The cellular location of overexpressed AhR was identified by immunofluorescence staining with FITC-conjugated antibodies and detected by Zeiss AXIO imager (40x). The nucleus was stained with Hoechst 33258. The image shows that the overexpressed AhR is mainly localized in the cytosol.

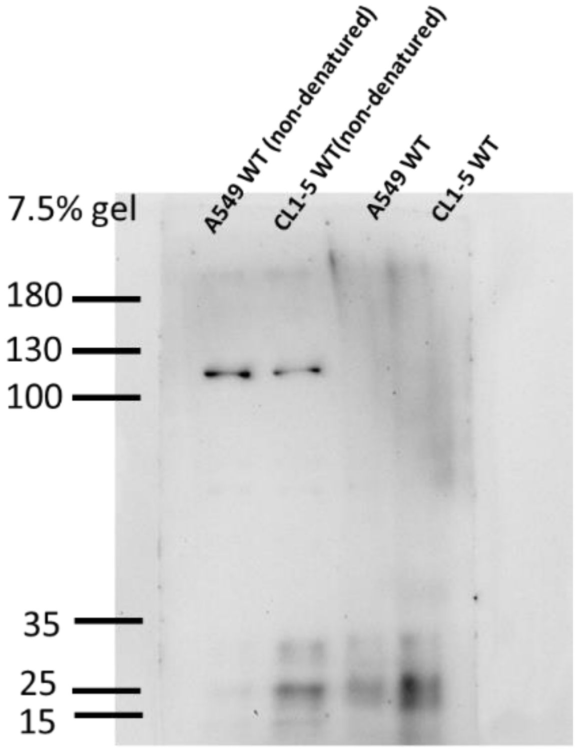

Supplementary Figure S10. The BNIP3 expression was detected by SDS PAGE with non-denatured manner. Protein level of A549 and CL1-5 cells were screen in non-denatured and normal(denatured) manner by Western blot. BNIP3 was captured by anti-BNIP3 which reveal a non-denatured band at 120kD; the normal sample reveal 25kD, respectively.

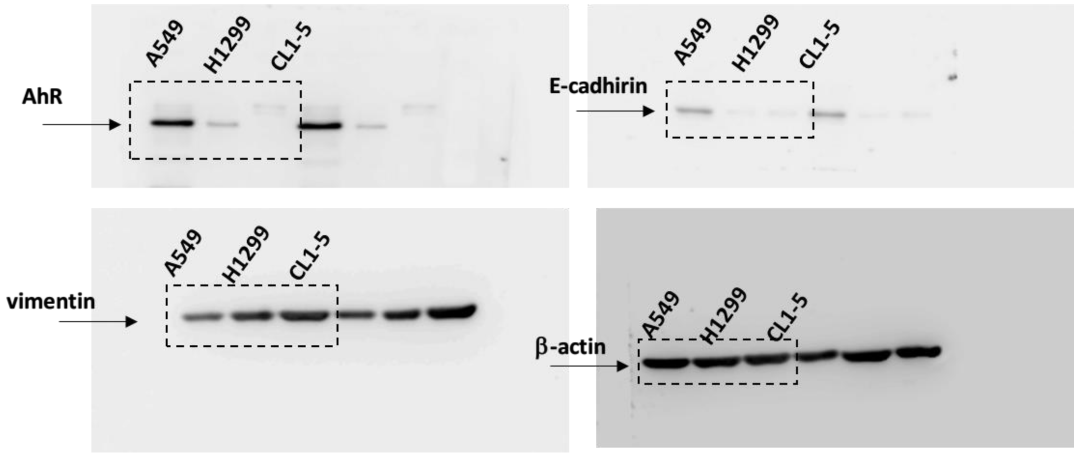

Supplementary Figure S11. Full-length image of figure 1b. Black dotted lines indicate the cropping locations.

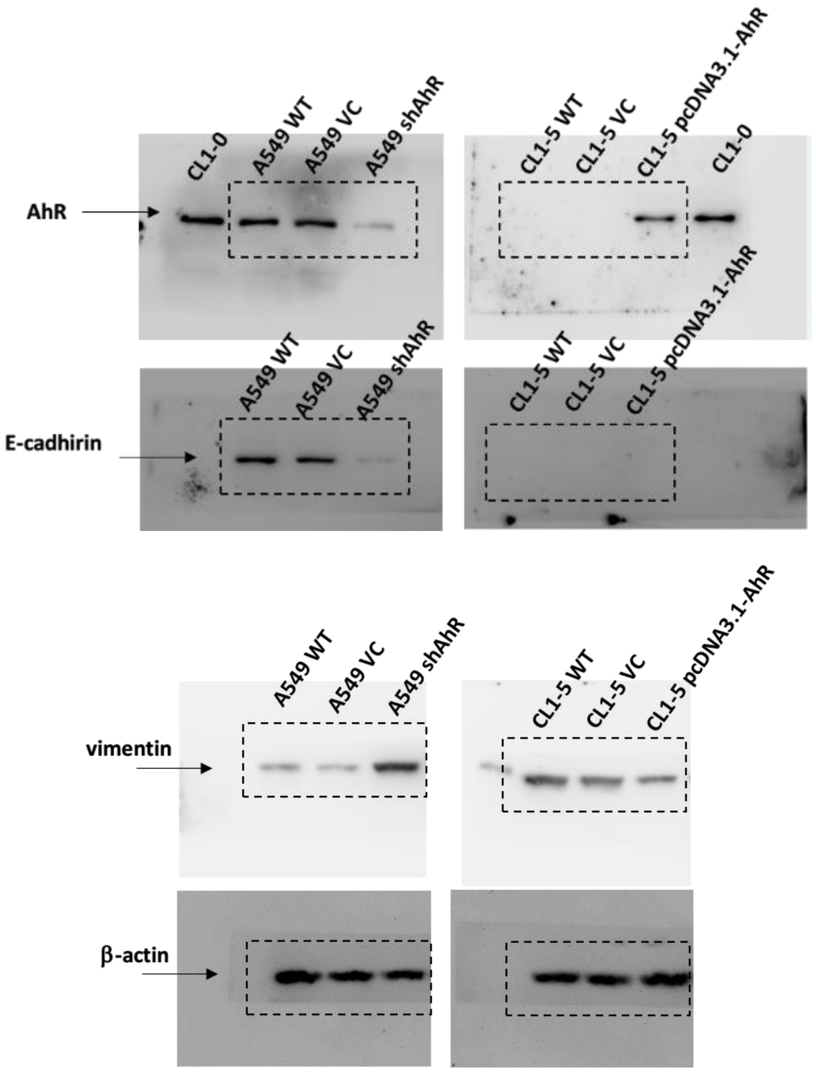

Supplementary Figure S12. Full-length image of figure 1c. Black dotted lines indicate the cropping locations.

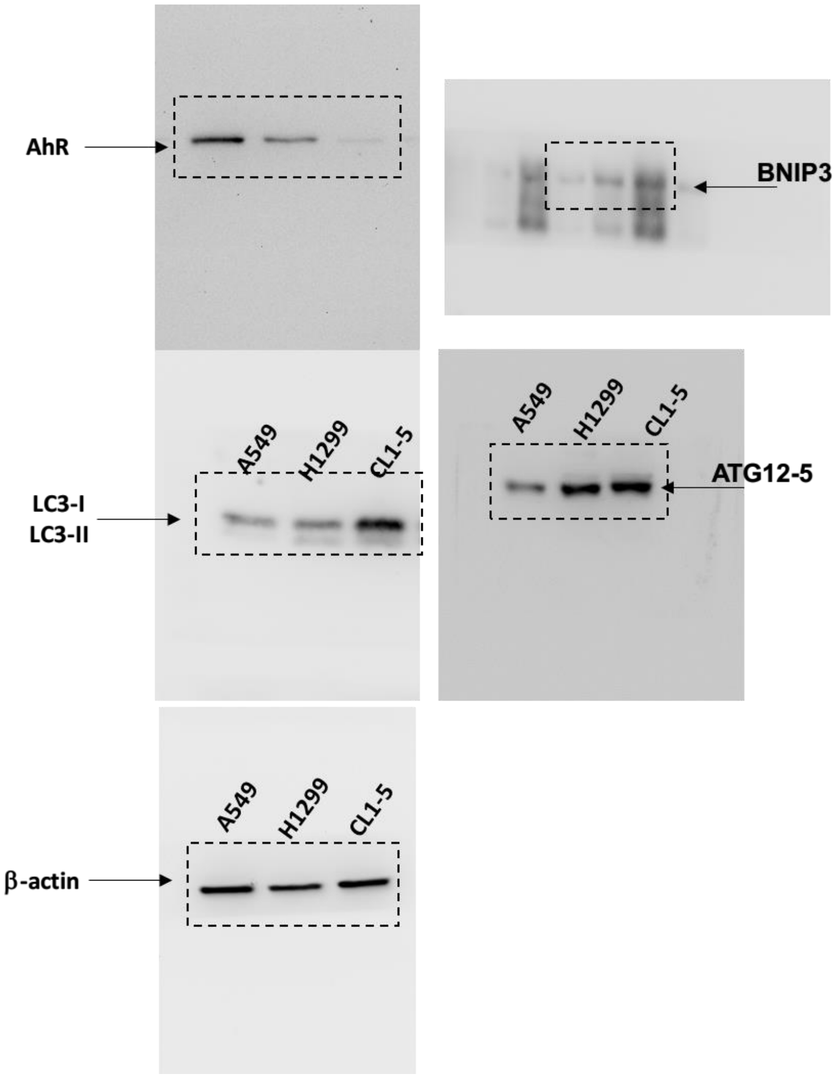

Supplementary Figure S13. Full-length image of figure 2a. Black dotted lines indicate the cropping locations.

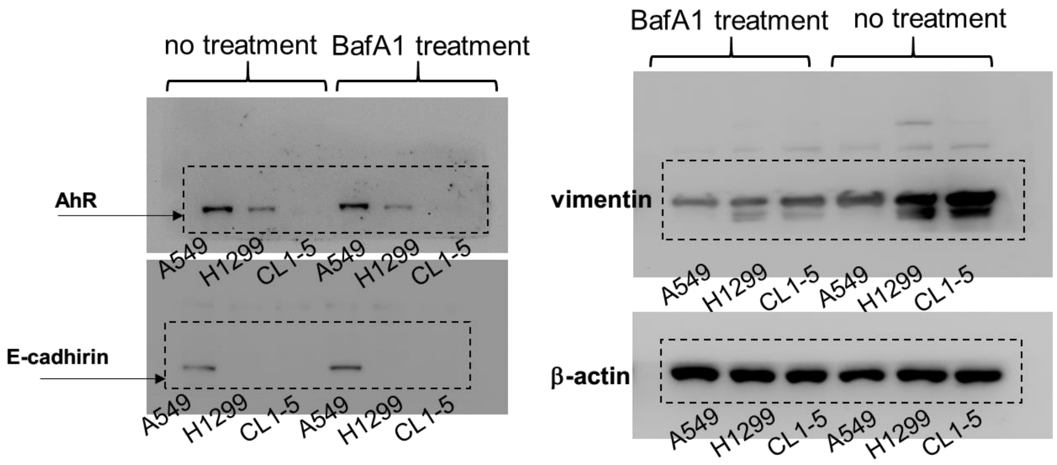

Supplementary Figure S14. Full-length image of figure 2c. Black dotted lines indicate the cropping locations.

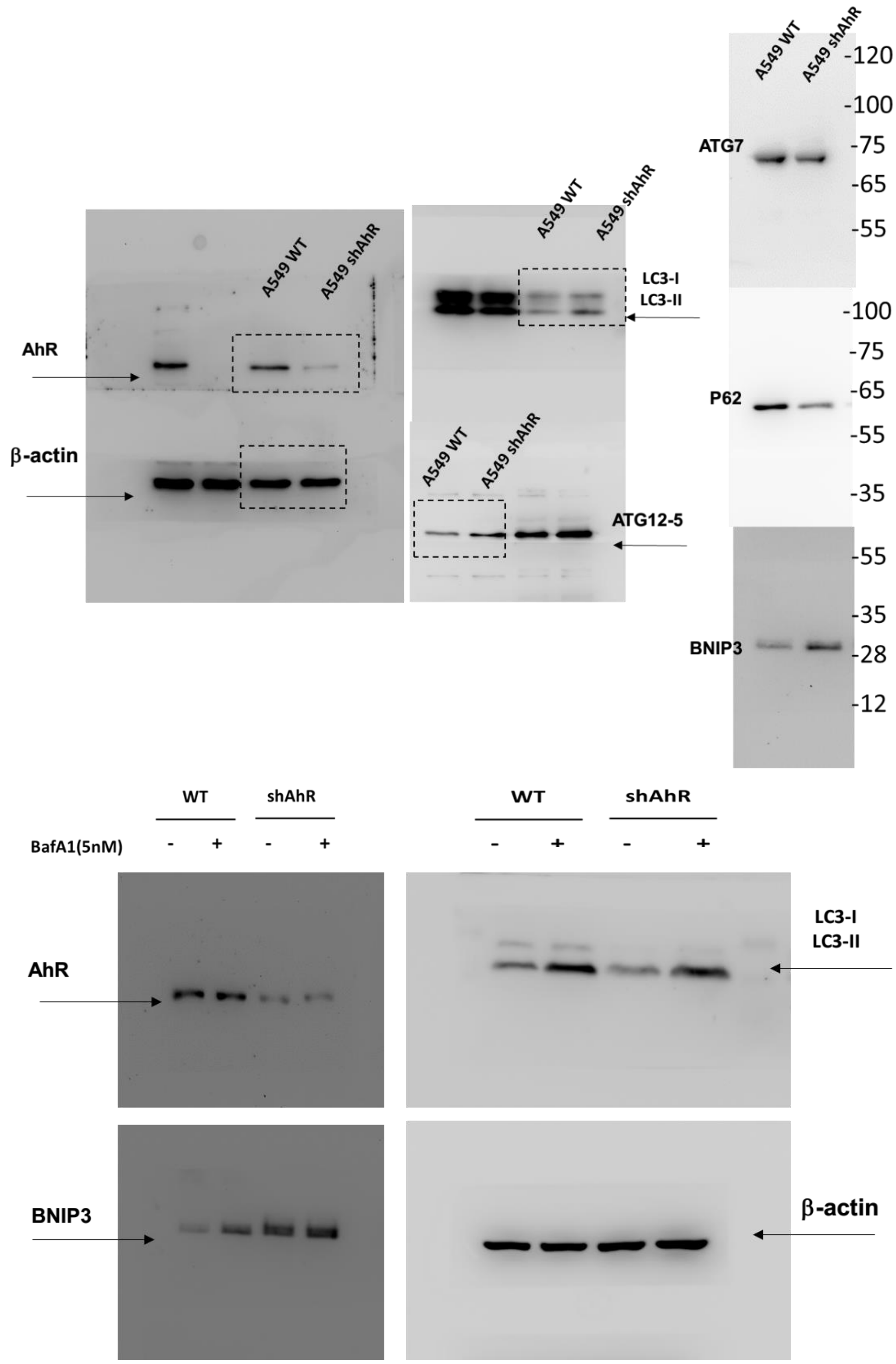

Supplementary Figure S15. Full-length image of figure 3. Black dotted lines indicate the cropping locations.

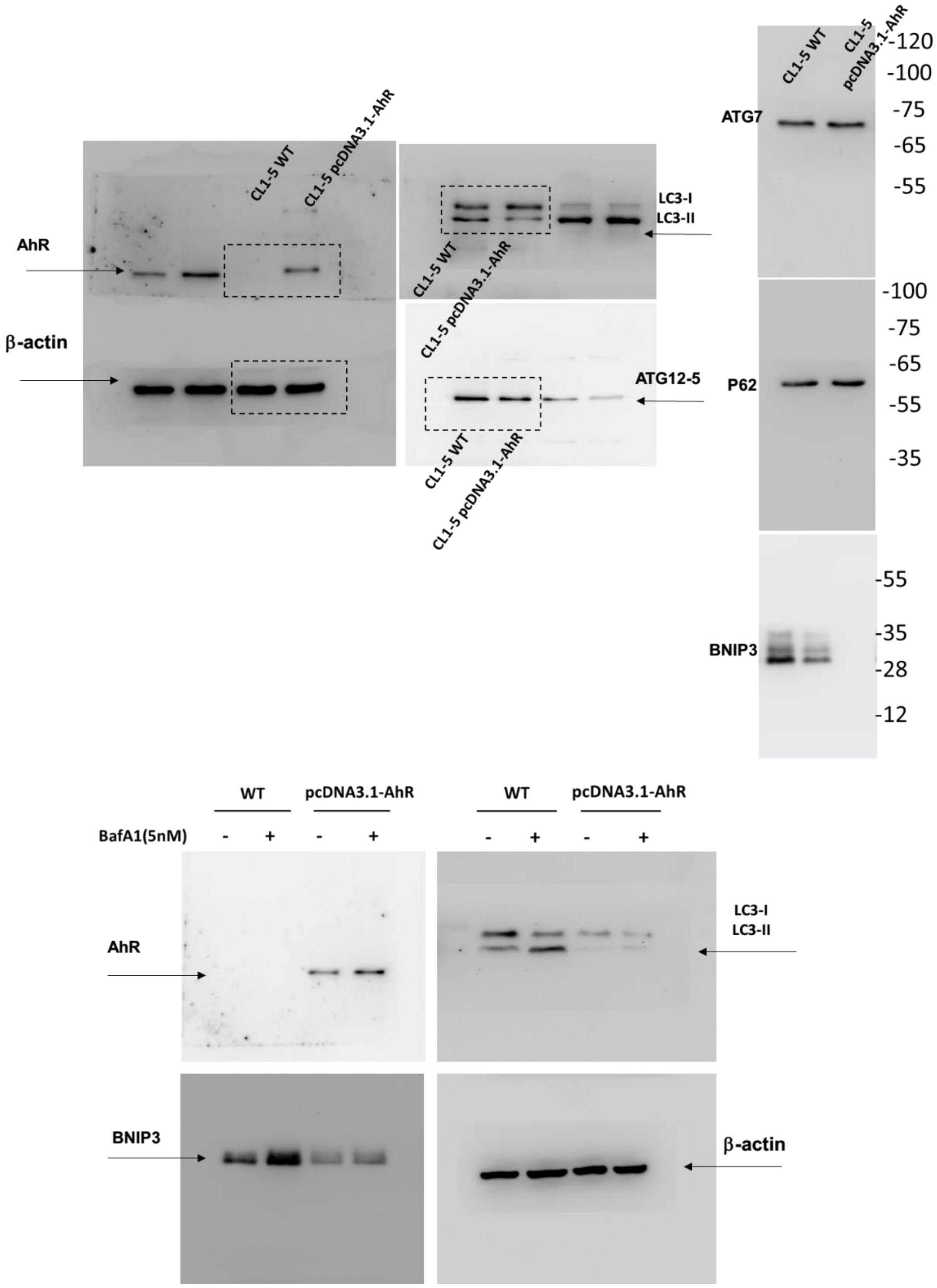

Supplementary Figure S16. Full-length image of figure 4. Black dotted lines indicate the cropping locations.

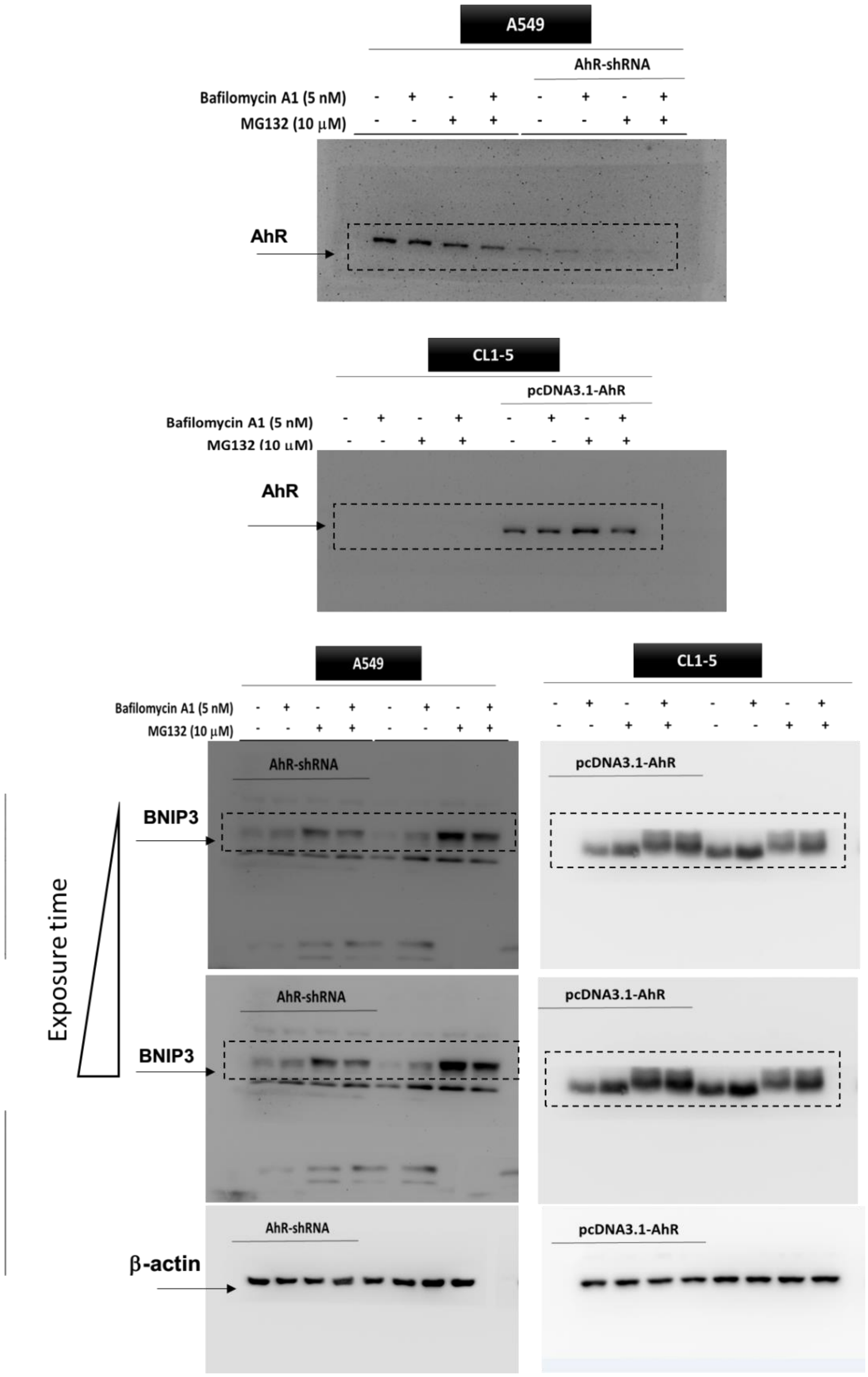

Supplementary Figure S17. Full-length image of figure 5. Black dotted lines indicate the cropping locations.

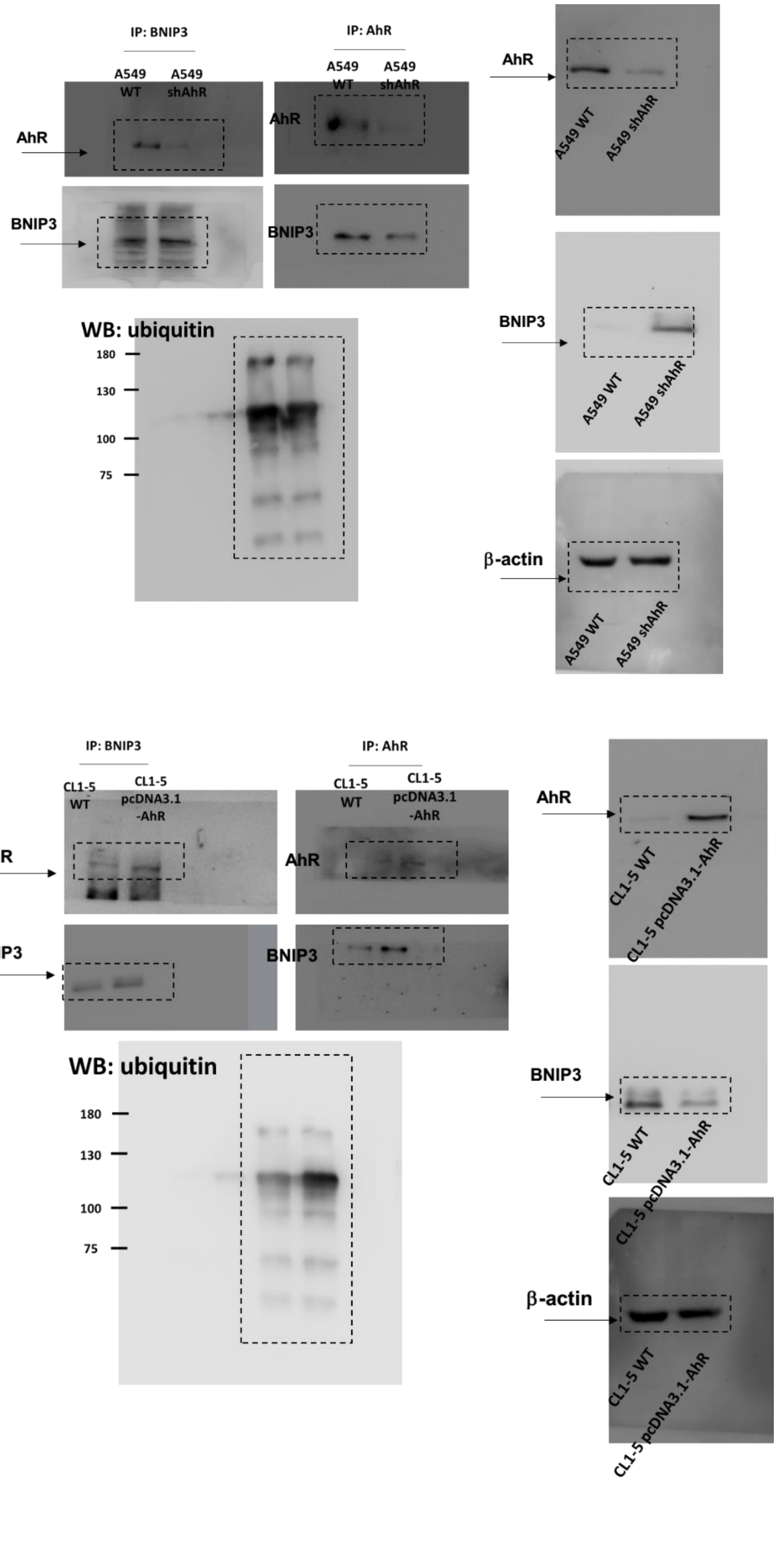

Supplementary Figure S18. Full-length image of figure 6. Black dotted lines indicate the cropping locations.
